# The watt or Kibble balance: a technique for implementing the new SI definition of the unit of mass

**DOI:** 10.1088/0026-1394/53/5/a46

**Published:** 2016

**Authors:** Ian A Robinson, Stephan Schlamminger

**Affiliations:** 1 National Physical Laboratory, Hampton Road, Teddington, Middlesex, TW11 0LW, UK; 2 National Institute of Standards and Technology (NIST), 100 Bureau Drive Stop 8171, Gaithersburg, MD 20899, USA

**Keywords:** kilogram, watt balance, redefinition, Kibble balance, Planck constant, mass measurement

## Abstract

The redefinition of the SI unit of mass in terms of a fixed value of the Planck constant has been made possible by the Kibble balance, previously known as the watt balance. Once the new definition has been adopted, the Kibble balance technique will permit the realisation of the mass unit over a range from milligrams to kilograms. We describe the theory underlying the Kibble balance and practical techniques required to construct such an instrument to relate a macroscopic physical mass to the Planck constant with an uncertainty, which is achievable at present, in the region of 2 parts in 10^8^. A number of Kibble balances have either been built or are under construction and we compare the principal features of these balances.

## 1. Introduction

Previously known as the moving-coil watt balance, the moving coil Kibble balance was invented at the National Physical Laboratory (NPL) by Bryan Kibble [1] in 1975 and it relates virtual mechanical and electrical power. Dr Kibble passed away in 2016 and the watt balance technique is now referred to as the Kibble balance technique in his honour. Originally, it was intended as a replacement for the current balance which realised the ampere from its definition in terms of the mechanical units [2, 3]. When combined with an SI ohm derived from the calculable capacitor [4], the Kibble balance can be used to realise the SI volt or SI ampere [5].

The discovery of the quantum Hall effect (QHE) by von Kitzing in 1980 [6], in conjunction with the previously discovered Josephson effect [7], led to the provision of highly stable representations of the ohm and volt, respectively [8]. The results from the National Institute of Standards and Technology (NIST) [9] and NPL Mark I [5] Kibble balance prior to 1990 contributed to the conventional values for the Josephson and von Klitzing constants K_J−90_ and R_K−90_, respectively. By fixing these constants in 1990, stable quantum representations of the volt and ohm were provided to the world. However, because they were fixed using data taken prior to 1990, they are close, but not equal, to the present best estimates of the SI volt and ohm.

The combination of these quantum effects enable electrical power to be measured in terms of the Planck constant *h* and frequency (counts per unit time). This enabled the Kibble balance to relate macroscopic mass to *h* with sufficiently low uncertainty to contemplate redefining the kilogram [10]. The Kibble balance would then represent a route to the realisation of the kilogram in the revised SI [11]. The existing proposals to revise the SI in 2018 would fix the values of *h* and the elementary charge *e* which would also fix the values of K_J_ and R_K_, within the SI, eliminating the need for K_J−90_ and R_K−90_ [12]. The Josephson and quantum Hall effects could then be used to realise the SI volt, the SI ohm and, in combination, the SI ampere.

### 1.1. Basic principles

**Figure 1.**
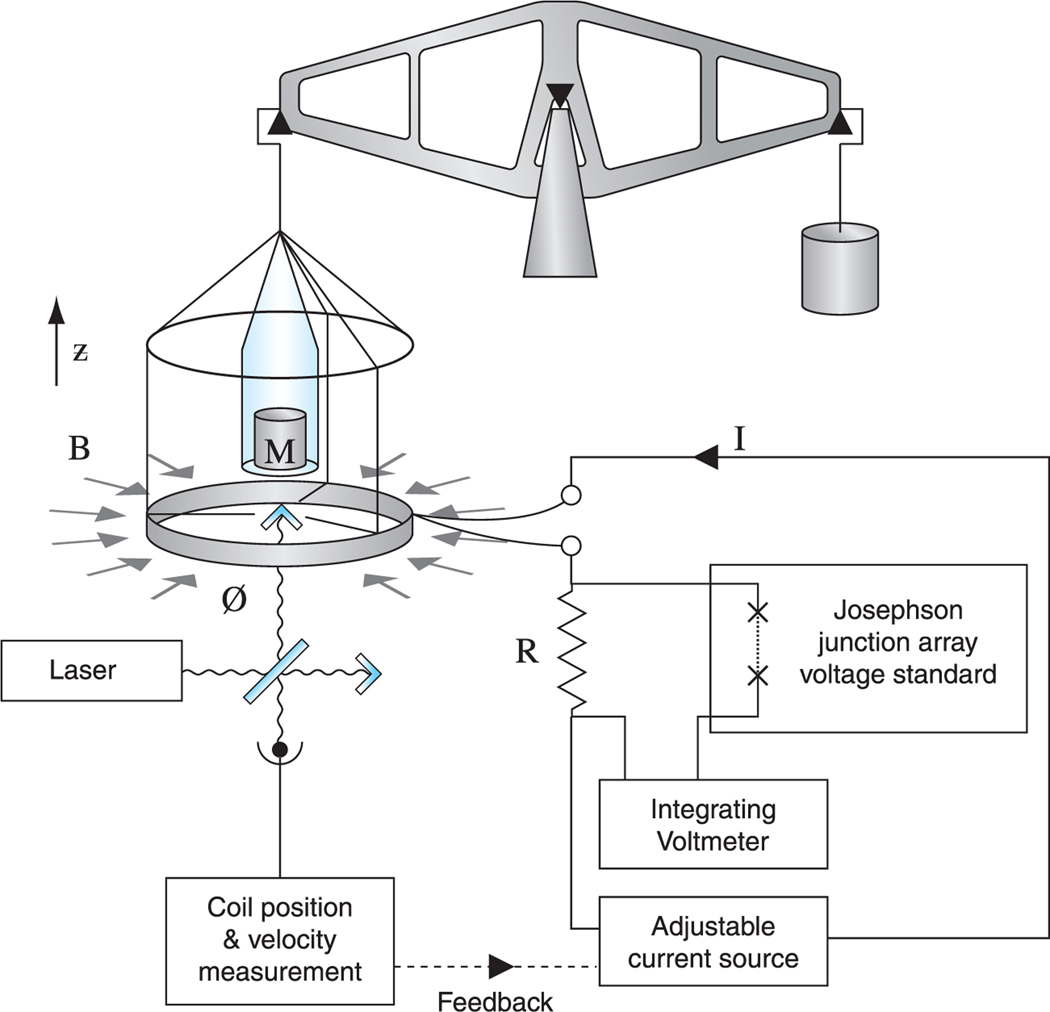
The Kibble balance in weighing mode.

**Figure 2.**
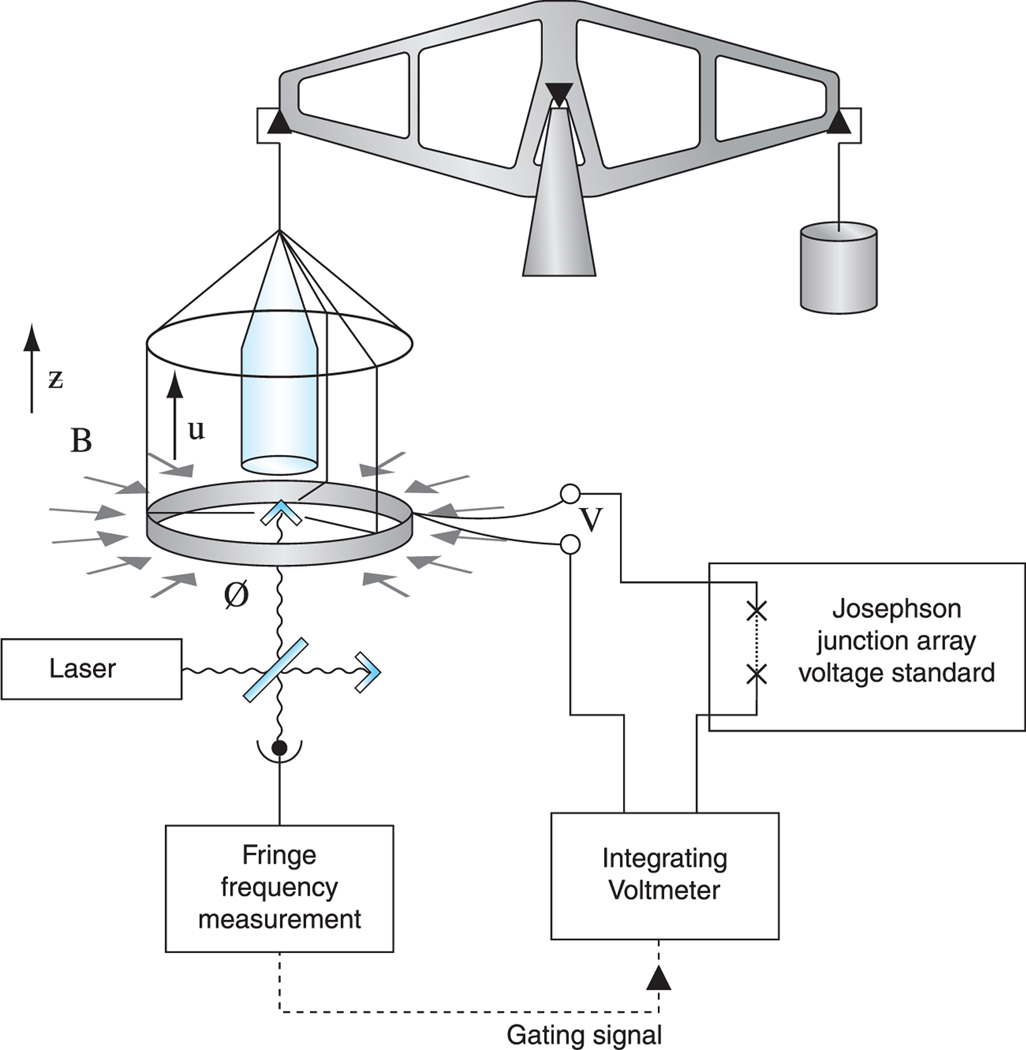
The Kibble balance in moving mode.

The Kibble balance consists of a coil of wire which is suspended from one arm of a balance and is placed in a strong magnetic field. The apparatus has two measuring modes: weighing mode and moving mode, which are illustrated in figures 1 and 2, respectively. In the weighing mode, the weight *M g* of a mass *M* is opposed by the vertical force *Bl I* generated by a current *I* flowing in a coil of wire of length *l* in a magnetic flux density *B* giving *M g* = *Bl I*. In the moving mode, the mass is removed and the coil is moved in the field with a velocity *u* which induces a voltage *V* = *Bl u* in the coil. If it is assumed that the quantity *Bl* is unchanged between the weighing and moving measurements, it can be eliminated giving:

(1) VI=Mgu.

As the measurements of force and current are separated from those of velocity and voltage, this expression equates virtual electrical to virtual mechanical power. Comparing virtual power eliminates the effects of energy loss mechanisms such as resistive losses, friction and eddy current losses, which would severely hamper a direct comparison of power. This makes it possible to achieve the extremely low overall uncertainty (of the order of 1 part in 10^8^) required for the redefinition of the kilogram.

The simple derivation of equation (1) ignores many issues, including the vector nature of the force generated by the coil, but in the next section we will show that equation (1) can be made to hold exactly under carefully defined circumstances.

## 2. The Kibble balance

### 2.1. Theory

Over the last few years, it has been observed that under some circumstances, the Kibble balance is immune to errors arising from secondary forces and torques, non-vertical motion of the coil, and electrical leakage effects [13, 14]. It was recognised in [14] that a Kibble balance which uses a common mechanism for both weighing and moving can act as a reciprocal system resulting in the cancellation of the above mentioned errors. The conditions required for these cancellations to occur in a virtual work system were derived in [15]. This theory covers the design of a range of Kibble balances and assumes that the motion of the coil is fully determined by the vertical velocity of the mass pan. The coil has coordinates (*x*, *y*, *z*) and its angles about the (*x*, *y*, *z*) axes are (*θ_x_*, *θ_y_*, *θ_z_*) and it is threaded by a magnetic flux Φ. The position of the mass pan along the vertical (*z*) axis is *z*′ and its vertical velocity is *u*_*z*′_.

The weighing mode of the balance is shown in figure 1, a current *I* is passed through the coil and the resultant of the forces and torques produced by the coil oppose the weight *Mg*. In the measuring phase corresponding to this mode of operation, the current *I* is measured. The equilibrium condition for the balance is:

(2) $$-Mg-I(\frac{\partial \text{Φ}}{\partial x}\frac{\partial x}{\partial z^{\prime }}+\frac{\partial \text{Φ}}{\partial y}\frac{\partial y}{\partial z^{\prime }}+\frac{\partial \text{Φ}}{\partial z}\frac{\partial z}{\partial z^{\prime }}+\frac{\partial \text{Φ}}{\partial \theta_{x}}\frac{\partial \theta_{x}}{\partial z^{\prime }}+\frac{\partial \text{Φ}}{\partial \theta_{y}}\frac{\partial \theta_{y}}{\partial z^{\prime }}+\frac{\partial \text{Φ}}{\partial \theta_{z}}\frac{\partial \theta_{z}}{\partial z^{\prime }})=0.$$

To measure the relevant properties of the coil and magnet, often referred to as the *Bl* product or geometric factor, the mass is removed, the current switched off and the apparatus is placed in moving mode as shown in figure 2. The mass carrier moves with a vertical velocity $u_{z^{\prime }}$. This motion causes the coil to move and rotate with velocities(*u_x_*, *u_y_*, *u_z_*) and angular velocities (*ω_x_*, *ω_y_*, *ω_z_*). These motions are related to the vertical velocity by:

(3) $$u_{x}=u_{z^{\prime }}\partial x/\partial z^{\prime }$$

(4) $$u_{y}=u_{z^{\prime }}\partial y/\partial z^{\prime }$$

(5) $$u_{z}=u_{z^{\prime }}\partial z/\partial z^{\prime }$$

(6) $$\omega_{x}=u_{z^{\prime }}\partial \theta_{x}/\partial z^{\prime }$$

(7) $$\omega_{y}=u_{z^{\prime }}\partial \theta_{y}/\partial z^{\prime }$$

(8) $$\omega_{z}=u_{z^{\prime }}\partial \theta_{z}/\partial z^{\prime }.$$

In the measuring phase associated with this mode, the velocity $u_{z^{\prime }}$ and the voltage *V* generated by the coil are measured giving:

(9) $$V=-(\frac{\partial \text{Φ}}{\partial x}u_{x}+\frac{\partial \text{Φ}}{\partial y}u_{y}+\frac{\partial \text{Φ}}{\partial z}u_{z}\;+\frac{\partial \text{Φ}}{\partial \theta_{x}}\omega_{x}+\frac{\partial \text{Φ}}{\partial \theta_{y}}\omega_{y}+\frac{\partial \text{Φ}}{\partial \theta_{z}}\omega_{z})$$

and, using (3)–(8),

(10) $$V=-u_{z^{\prime }}(\frac{\partial \text{Φ}}{\partial x}\frac{\partial x}{\partial z^{\prime }}+\frac{\partial \text{Φ}}{\partial y}\frac{\partial y}{\partial z^{\prime }}+\frac{\partial \text{Φ}}{\partial z}\frac{\partial z}{\partial z^{\prime }}\;\;+\frac{\partial \text{Φ}}{\partial \theta_{x}}\frac{\partial \theta_{x}}{\partial z^{\prime }}+\frac{\partial \text{Φ}}{\partial \theta_{y}}\frac{\partial \theta_{y}}{\partial z^{\prime }}+\frac{\partial \text{Φ}}{\partial \theta_{z}}\frac{\partial \theta_{z}}{\partial z^{\prime }}).$$

If the only significant forces or torques are produced by gravity *g* or the interaction of current and magnetic flux and the values of the partial derivatives in (3)–(8) and the spacial derivatives of Φ do not change during and between moving and weighing modes (the stability conditions), then it is possible to combine (2) and (10) to give the exact equation

(11) $$Mgu_{z^{\prime }}=VI.$$

If the stability conditions are met, the Kibble balance can relate its principal measurands without the need for precise alignment. However, if the balance is not well aligned, motions caused by misaligned forces and torques can invalidate the stability conditions. To ensure that these conditions are met, all existing Kibble balances are carefully aligned. A technique has been proposed to overcome these limitations [16] which should simplify the construction and operation of Kibble balances and is described in section 2.2.5. The existing NIST, NPL/National Research Council (NRC) and Measurement Standards Laboratory (MSL) Kibble balances use common weighing and moving mechanisms employing pressure or knife-edge balances which require care to reach the required weighing sensitivity. An alternative is a flexure-based balance which is extremely sensitive and does not display the hysteresis problems inherent in knife edge balances. However, existing flexure-based balances cannot generate the large excursions required in the moving mode of the Kibble balance and, to overcome this problem, it is usually necessary to adopt a separate mechanism for moving the coil which can make the apparatus sensitive to parasitic forces, torques and motions as illustrated below.

If it is assumed that the weighing mechanism in a flexure-based balance is sensitive only to vertical forces then equation (2) becomes:

(12) Mg=−I∂Φ/∂z.

The other terms in equation (2) can be represented in terms of torques (${\text{Γ}}_{x}$, ${\text{Γ}}_{y}$, ${\text{Γ}}_{z}$) and horizontal forces (*F_x_*, *F_y_*) :

(13) $$F_{x}=-I\;\partial \text{Φ}/\partial x$$

(14) $$F_{y}=-I\;\partial \text{Φ}/\partial y$$

(15) $${\text{Γ}}_{x}=-I\;\partial \text{Φ}/\partial \theta_{x}$$

(16) $${\text{Γ}}_{y}=-I\;\partial \text{Φ}/\partial \theta_{y}$$

(17) $${\text{Γ}}_{z}=-I\;\partial \text{Φ}/\partial \theta_{z}$$

using (13)–(17) equation (9) becomes

(18) $$VI=(F_{x}u_{x}+F_{y}u_{y}+Mgu_{z}\;\;+{\text{Γ}}_{x}\omega_{x}+{\text{Γ}}_{y}\omega_{y}+{\text{Γ}}_{z}\omega_{z}).$$

or

(19) $$VI=Mgu_{z}(1+\frac{F_{x}u_{x}}{Mgu_{z}}+\frac{F_{y}u_{y}}{Mgu_{z}}\;\;+\frac{{\text{Γ}}_{x}\omega_{x}}{Mgu_{z}}+\frac{{\text{Γ}}_{y}\omega_{y}}{Mgu_{z}}+\frac{{\text{Γ}}_{z}\omega_{z}}{Mgu_{z}}).$$

Under these circumstances, the Kibble balance equation is no longer exact and the power ratios in the bracket of equation (19) represent fractional errors which must be eliminated. This is achieved by careful alignment of the balance to reduce the unwanted forces, torques and motions to levels where their products are either less than 1 part in 10^9^ of the measured virtual power *M g u_z_* or are known with this uncertainty and a correction can then be applied.

The techniques used to align Kibble balances apply to all existing balances and are discussed in section 3.8.

To relate the kilogram to fundamental constants, the virtual electrical power *VI* is measured using the quantum Hall effect (QHE) and the Josephson effect. The Josephson effect allows the voltage *V* to be determined in terms of a measured microwave frequency *f* as V=hf/2e. The QHE generates a resistance $R=h/ne^{2}$ where *n* is a quantum number. By suitable scaling and in combination with the Josephson effect ($V^{\prime }=hf^{\prime }/2e$) the current $I=V^{\prime }/R=nef^{\prime }/2$ can be measured in terms of *e* and frequency *f*′. The elementary charge *e* cancels in the product of voltage and current $VI=hff^{\prime }n/4$ and so, via equation (11) or (19), mass can then be related to the Planck constant *h*, the metre and the second.

### 2.2. Types of Kibble and joule balances

Since the original invention of the Kibble balance, a number of different balances have been described and built by laboratories around the world. Mostly, they differ in the details of their construction such as: the size of the balance, mechanisms used for moving the coil, weighing the mass and other details. However, there are some more significant variations which can be categorised into a relatively small number of types. This section describes the major variant types and the existing watt balances that exemplify each type. To conserve space, the descriptions have been limited to the latest version of each existing balance but, if previous versions exist, a reference has been made to them.

#### 2.2.1. Conventional two-mode two measurement phase Kibble balances.

As described in section 1, the original Kibble balance [3] has two modes, a weighing mode and a moving mode, each of which is associated with a measuring phase which collects either weighing or moving data. The Kibble balances listed below, in alphabetical order of the institutes’ acronyms, use this operating principle.

##### Korea Research Institute of Standards and Science (KRISS), Korea:

The KRISS Kibble balance [17] uses a circular coil in the radial field of a permanent magnet. The coil is suspended from a commercial weighing cell and both are guided in moving mode by the ‘piston in a cylinder’ technique pioneered by MSL (see below). The balance operates in vacuum.

##### Laboratoire National de Métrologie et d’Essais (LNE), France:

The balance and coil of the LNE Kibble balance are supported by a large flexure bearing [18, 19] which is designed to provide accurate vertical movement. The balance is a custom made flexure balance which supports the coil and is locked during the moving phase. The circular coil is placed in the radial field of a permanent magnet. The apparatus can be operated in vacuum.

##### Federal Institute of Metrology (METAS), Switzerland:

Researchers at METAS have built two Kibble balances. The first is described in [20, 21]. The second, METAS Mark II [22], uses an optimised flexure stage [23] to move a customised commercial weighing cell and the circular coil. The magnet generates a radial field and is temperature compensated using a magnetic shunt. The balance can operate in vacuum.

##### Measurement Standard Laboratory of New Zealand (MSL):

Researchers at MSL use a pressure balance for both the weighing and moving modes. The cylinder of the pressure balance provides guidance for the coil in moving mode and the normal operation of the pressure balance allows the comparison of coil force with the weight of the mass. To minimise the effects of ground vibration, they intend to use an oscillatory motion of the coil in the moving mode [24] rather than gathering data at a uniform velocity as adopted by other balances.

##### National Institute of Metrology (NIM), China:

The original NIM joule balance technique is described in section 2.2.2. Recently the apparatus has been changed to use a conventional magnet/electromagnet which makes the original mutual inductance measurement technique difficult. To measure the geometric factor, they have adopted a variant of the methods used in the moving phase of the Kibble balance (see section 3.3). Almost all the techniques described in section 3 are relevant to the operation of this form of joule balance; where there are significant differences these have been pointed out in the text.

##### National Institute of Standards and Technology (NIST), USA:

The first three NIST Kibble balances used electromagnets, versions two and three a superconducting magnet [25]. The NIST-4 balance uses a permanent magnet [26] and a circular coil. The coil is suspended from a wheel balance using a band of fine titanium wires. It operates in vacuum.

##### National Physical Laboratory (NPL), UK:

NPL has made two Kibble balances the Mark I is described in [5]. The Mark II balance employs a circular coil on a glass former suspended in the field of a permanent magnet. The coil is suspended from the balance beam used in the Mark I apparatus which employs knife edges. The balance operates in vacuum.

##### National Research Council (NRC), Canada:

The NPL Mark II Kibble balance was shipped to NRC in 2009. Modifications were made to the mass lift [27] and the coil support system to eliminate problems identified by NPL prior to shipment. Further modifications have been made to reduce the noise of the moving measurements and allow alignments to be made while the apparatus is under vacuum.

#### 2.2.2. The original joule balance.

##### National Institute of Metrology (NIM), China:

The original NIM joule balance design [28] replaced the moving mode of the Kibble balance with measurements of mutual inductance [29] between two stationary coils: the coil which generates the field for the weighing mode (the exciting coil) and the movable coil [30]. Each measurement is made by applying a linear current ramp to the field generating coil which induces a constant voltage in the stationary movable coil, similar to [31]. This measurement is repeated for different positions of the movable coil. Weighings are performed with constant currents in the exciting and movable coils. The resulting equation relates virtual energies which gives rise to the name of the technique. The first generation joule balance reported results in 2014 [28] but heating effects and problems with mutual inductance measurement have concentrated future work onto its successor which is described in section 2.2.1 and no longer measures mutual inductance.

#### 2.2.3. Single-mode one measurement phase Kibble balances.

##### Bureau International des Poids et Mesures (BIPM):

The BIPM introduced a variation to the Kibble balance technique [32] whereby the two measurement modes are combined into one and the two measurement phases are carried out simultaneously. This has the advantage that, as the weighing current is always flowing in the coil, changes in the magnetic field of the magnet do not affect the measurement but, in their original proposal, a superconducting coil [33] was required to ensure that the moving voltage could be measured accurately with the weighing current flowing in the coil. BIPM are presently working on a room temperature implementation of the technique.

#### 2.2.4. Moving magnet balances.

##### Ulusal Metroloji Enstitüsü (UME), Turkey:

Recently, researchers at UME have proposed a Kibble balance where the magnet is moved and oscillates about an equilibrium position [34]. This technique separates the signal in weighing mode and velocity mode in frequency space. The weighing current is applied and measured mostly at zero frequency, while the electromotive force is generated mostly at the oscillation frequency. If the field is not perfectly uniform, the signals will spill over in multiples of the oscillation frequency. In principle, however, it is possible to separate the resistive voltage drop across the coil from the induced voltage. So far there is only a white paper on this idea.

#### 2.2.5. Single-mode two measurement phase watt balances.

##### National Physical Laboratory (NPL), UK:

Recently, NPL has proposed a technique [16] to make a Kibble balance which does not require precise coil alignment. The technique employs a variant of the BIPM single weighing/moving mode but the measurement phases are separated in the manner of the original Kibble balance. By treating the mass raised and mass lowered states of the balance as two independent Kibble balances, the theory introduced in section 2.1 can be applied to eliminate the need for precise alignments.

The uncertainties achievable using this technique should be equivalent to those achieved by existing Kibble balances but the advantage will be in simplified construction and operation. Once the kilogram has been redefined, more laboratories will wish to have an independent realisation of the unit of mass which will enable them to contribute to a worldwide consensus mass scale [35]. Cost, both to acquire and operate, is becoming an increasingly important concern and this new idea may help to address these issues.

## 3. Design and operation of Kibble balances

### 3.1. Magnets

#### 3.1.1. Source of the magnetic field and flux guides.

In general, a magnetic field can be produced by a polarized ferromagnetic material or by a current. In the latter case, the current can flow through either a conventional or a superconducting coil. All three types of sources have been used to build Kibble balances. However, the most recent designs all use permanent magnets to source the field. Because designs with permanent magnets are, generally speaking, cheaper, simpler, and easier to use. The main disadvantage of systems with permanent magnets is the fact, that the magnetic flux density cannot be varied over a large range. It can be useful for the experimenter to change the magnitude of the field to study systematic effects. Electromagnets on the other hand can be easily changed to different values by adjusting the current in the coils. As a consequence, they require a feedback system to sufficiently stabilize the current during normal operations. This is not a trivial task, if a relative field stability of 10^−8^ is required.

Regardless of the source of the magnetic field, the magnet system can be designed with or without a yoke. A soft-iron yoke concentrates the magnetic flux from the source into a small volume which is swept through by the coil in moving mode. Magnet systems with yokes use the magnetic energy very efficiently. Because the magnetic energy is directed to the coil volume, referred to as the gap and very little magnetic energy is wasted to other regions. In yoke-less systems, the magnetic energy is usually spread out over a large volume.

A disadvantage of the yoke is that another, magnetically non-linear, material is introduced to the system. Special attention needs to be paid to the effect of the weighing current on the yoke [36, 37].

Halbach arrays [38] are an alternative to using a yoke to achieve a magnetic field in a confined region of space and mitigate the disadvantage of having to introduce a magnetically non-linear material.

A big advantage of a yoke, that Halbach arrays cannot deliver, is magnetic shielding. If designed correctly, a yoke can effectively shield the coil from varying external magnetic fields. The yoke can also shield the working mass from the magnetic field of the permanent magnet. Furthermore, the yoke also allows the magnetic field to be shaped. For example, the shape of the gap can be modified by precision grinding, allowing the magnet designer to manipulate the vertical profile of the magnetic flux density.

#### 3.1.2. The shape and direction of the field.

The direction of the magnetic force is given by the cross product of the current and the magnetic flux density for each line element along the wire of the coil. Hence, it is immediately clear, in order to produce a vertical force, the field has to be horizontal.

The most efficient use of the wire is achieved by coiling it up in a circle with a vertical area vector and by using a field that is perpendicular to the wire at each point, i.e. a radial field.

A beautiful cancellation of the dependence of the geometric factor from the size of the coil, and hence the temperature, can be achieved if the magnetic flux density falls off as 1/*r*. This idea is credited to Olsen [39]. In this case, the product of the flux density and the wire length remains the same independent of thermal expansion and contraction. For example, as the radius of the coil grows due to thermal expansion, the magnetic flux density encountered by the coil is reduced by the same relative amount as the wire length is increased.

Ideally, the geometric factor remains unchanged over the length of the coil sweep, i.e. the magnetic flux density is independent of *z*. In this case, for a fixed velocity, the induced voltage remains constant and is easy to measure. Also, the precise location of the weighing position does not matter. A constant magnetic flux density with vertical position is practically impossible to achieve due to the fringing fields at the ends of the gap. Typically the field varies by a part in 10^4^ over the 10’s of mm swept by the coil and the designer of the magnet systems tries to keep this field variation as small as possible.

### 3.2. Considerations for the magnet system

Every current Kibble balance uses a magnetic field for its operation. The purpose of the magnet system is to provide the magnetic field at the weighing position of the coil and a few centimetres above and below this position. As discussed above, in the ideal case, the field is purely radial at the weighing position, i.e. it has no vertical component *B_z_* = 0 and the radial component *B_r_* is only weakly dependent of the vertical coil position *z*, in the best case $\partial B_{r}/\partial z=0$.

Today, researchers around the world agree that a permanent magnet system is the most efficient way to generate the magnetic field. A permanent magnet system is typically constructed of two different materials: an active magnetic material, typically Samarium–Cobalt, and a material that guides the flux, very often mild steel. The magnetic flux is concentrated into an annular gap that houses the coil. Currently all existing Kibble balances use one or more circular coils with a vertical axis of symmetry. The NPL/NRC Kibble balance uses two circular coils mounted on a single former. The two coils are connected in series opposition and are vertically displaced in two different air gaps.

**Figure 3.**
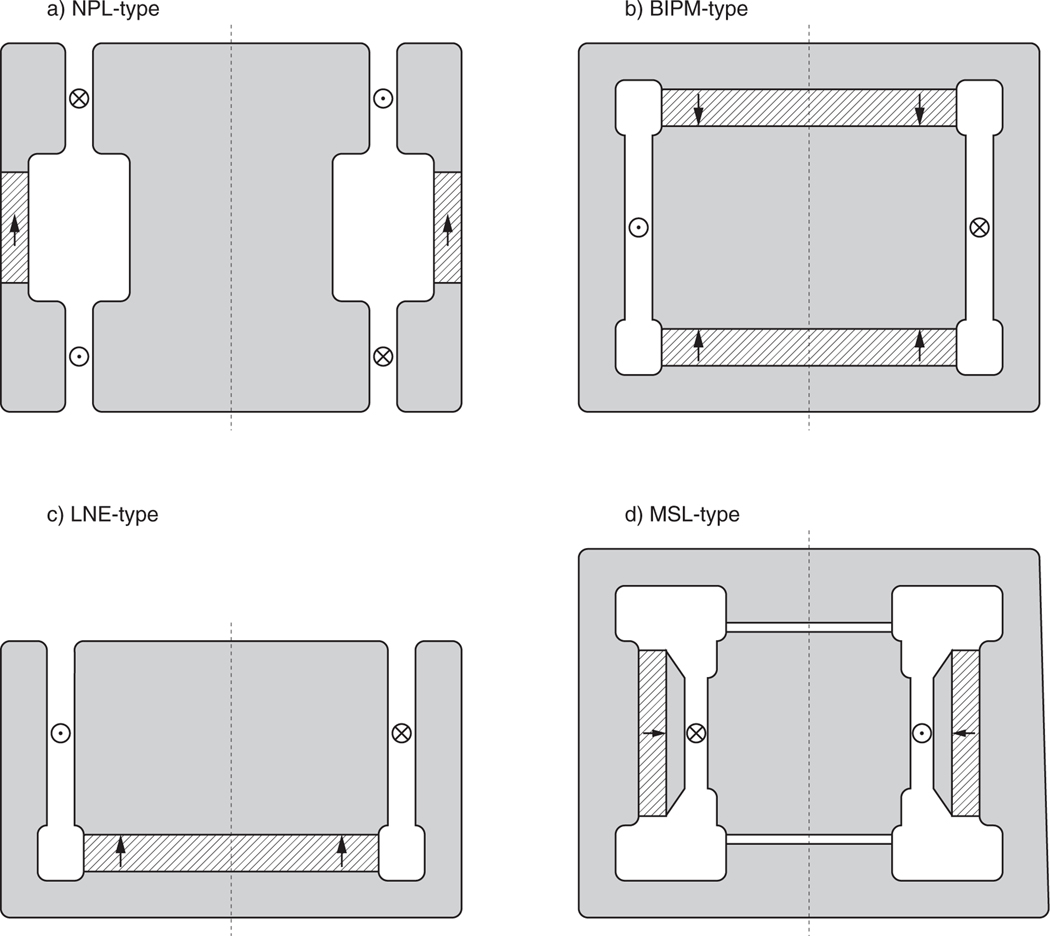
Schematic drawings of the four permanent magnet designs used in Kibble balances. All magnets exhibit rotational symmetry around the dashed line in the centre. The grey shaded parts concentrate the flux and are typically manufactured of mild steel. The hatched parts represent the active magnetic material. The arrows indicate a possible direction of the magnetic polarization of the material. (a) NPL-type. (b) BIPM-type. (c) LNE-type. (d) MSL-type.

Four different arrangements of the yoke and the active magnetic material are in use today, shown in figure 3. The two designs pictured on the left of figure 3, the NPL-design and the LNE-design, allow access to the complete gap from the top. The coil can easily be inserted into the gap. In the BIPM and MSL designs, the coil is completely surrounded by the magnet; therefore, holes in the top or bottom yoke piece allow the penetration of rods that connect the coil to the balance and stirrup system. After the coil has been placed into the magnet-system during construction, it needs to be closed. Different strategies are used to close the magnet. The top plate of the original BIPM magnet consists of sectors. After the coil is placed in the gap, the sectors are put in place, completing the magnetic circuit on the top. Using sectors instead of a monolithic top plate keeps the magnetic forces at a manageable level. The researchers at NIST completely split the magnet in two parts, insert the coil and rejoin the magnet. This procedure needs a dedicated sturdy device because the magnetic forces can be quite large, in excess of 10 kN. The BIPM and MSL designs achieve a flat field at the weighing position because this position is in a horizontal plane of mirror symmetry.

In the NPL and LNE design, a flat field can be achieved by carefully engineering the width of the gap as a function of vertical position [40].

Besides the arrangement of the active magnetic material and yoke, other design parameters are: the useful height of the gap, the width of the gap, the mean radius of the coil and the strength of the magnetic flux density at the coil position. Table 1 summarizes the design choices that have been made by nine groups. Remarkably, the design parameters only vary within small ranges: the magnetic flux density at the mean radius of the coil varies from 0.42 T to 0.95 T. The gap width ranges from 8 mm to 30 mm. The smallest usable height of the coil is 34 mm, the largest 100 mm. The nominal coil radius is between 72 mm and 215 mm. The geometric factor, Bl=∂Φ/∂z, approximately the product of the coil’s nominal circumference, the magnetic flux density at this position, and the number of turns. For the available data, the geometric factor ranges from 300 T m to 1250 T m.

The flatness of the field at the weighing position, $\partial B_{r}/{\partial z|}_{z=0}$, is an important concern. Typically two measurements are performed in the weighing mode, named mass off and mass on. Depending on the compliance of the coil support and the details of the balance control, the coil can be at two different vertical positions for each of these two measurements. The substitution measurement that is carried out during force mode can be written in one equation as

(20) $$I_{\text{Off}}lB\left(z_{\text{Off}}\right)=I_{\text{On}}lB\left(z_{\text{On}}\right)-Mg$$

Instead of using the position of the coil coordinates during mass on and mass off, we use the difference and mean values, i.e. $\bar{z}=\frac{1}{2}\left(z_{\text{On}}+z_{\text{Off}}\right)$ and $\text{Δ}z=\frac{1}{2}\left(z_{\text{On}}-z_{\text{Off}}\right)$. A similar transformation can be made for the currents, $I_{\text{A}}=\frac{1}{2}\left(I_{\text{On}}-I_{\text{Off}}\right)$ and $\delta I=\frac{1}{2}\left(I_{\text{On}}+I_{\text{Off}}\right)$. Here, *I*_A_, the current amplitude, is a large positive number and *δI* is a small number which indicates how symmetric the mass on and mass off currents are about zero current. Replacing these variables and solving equation (20) for the weight of the test mass yields after expanding the result in a Taylor series up to second order in Δz and δI,

(21) $$Mg\approx 2I_{\text{A}}lB(\bar{z})\left(1+\frac{\delta I}{I_{\text{A}}}\frac{{\text{Δ}z\frac{\partial B}{\partial z}|}_{z=\bar{z}}}{B(\bar{z})}+\frac{1}{2}\frac{{{\left(\text{Δ}z\right)}^{2}\frac{\partial^{2}B}{\partial z^{2}}|}_{z=\bar{z}}}{B(\bar{z})}\right).$$

The second term in the parenthesis on the right side of the above equation can be made zero by adjusting the currents such that they are equal and opposite, *δI* = 0. In traditional Kibble balances, the current offset *δI* is adjusted by adding or removing a small amount of mass on the tare side of the balance.

Assuming a finite Δ*z*, the third term can only be made zero if the second derivative of the radial field with respect to *z* is zero. The effect of the third term is usually negligible as it scales with the square of Δ*z*, which is small. Publications of watt balances report typical relative changes of the magnetic flux over the height of the gap to be about 10^−4^. Assuming a quadratic profile and a gap height of 8 cm, $\partial^{2}B/\partial z^{2}/B$ is 0.125 m^−2^. Combining this value with Δ*z* = 10 μm yields 6.3 × 10^−12^ for the third term in the parentheses on the right side of equation (21). This number is more than three orders of magnitude smaller than the typical uncertainties achieved by Kibble balances. In conclusion, the field flatness does not play a major role in the weighing mode as long as the weighing currents are symmetric about zero.

A flat field is also desired for the moving mode. The electrical measurements benefit if the induced electro motive force (EMF) stays constant as a function of time. In this case, an equal and opposite voltage can be added to the EMF and a null measurement with high gain can be made. If the profile is flat, it is easy to achieve a constant EMF by moving the coil with constant velocity. If the field changes significantly over the region where measurements take place, the coil velocity may have to be varied slightly to maintain a constant EMF. This is not difficult but the variation can often be absorbed by the dynamic range of the voltmeter used for the measurement.

#### 3.2.1. Effect of the weighing current on the magnetic flux density.

One important assumption for the two-mode two-measurement-phase Kibble balances is that the geometric factor is the same in the weighing and the moving mode. However, in the weighing mode the coil carries a current and in the moving mode it does not. The current causes ohmic heating and a magnetic field. Both effects can change the *B l* between the modes.

A popular model for the dependence of the magnetic field on the current has been introduced by researchers at NPL [46]. The magnetic flux density is written as

(22) $$B(I)=B_{o}\left(1+\alpha I+\beta I^{2}\right).$$

Rewriting equation (20) to reflect the change in *B* as a function of current is

(23) $$I_{\text{Off}}lB\left(I_{\text{Off}}\right)=I_{\text{On}}lB\left(I_{\text{On}}\right)-Mg.$$

Using $I_{\text{On}}=\delta I+I_{\text{A}}$ and $I_{\text{Off}}=\delta I-I_{\text{A}}$ yields

(24) $$Mg=2lB_{o}I_{\text{A}}\left(1+2\alpha \delta I+3\beta \delta I^{2}+\beta I_{\text{A}}^{2}\right).$$

There are three corrections terms to the unbiased term, 2*B_o_I*_A_. The first two terms are proportional to the current asymmetry. These vanish, if the weighing current for the mass off state is exactly equal and opposite to the current in the mass on state. The final term, *βI*_A_^2^ is proportional to the current amplitude squared. This term can introduce a serious bias to the Kibble balance experiment but its magnitude can be estimated by using test masses with different mass values. For example, the relative size of this effect would quadruple, if a 0.5 kg test mass is replaced by a 1 kg test mass. Note, introducing another odd term in equation (22) yields another term proportional to *δI*. Only even powers of *I* produce bias terms which depend on *I*_A_.

While the methods above provide an experimental way to estimate the magnet non-linearity, they do not provide a reason for the effect. Several possibilities exist to provide an explanation:

##### Demagnetization of the rare earth magnets.

The current in the coil adds or subtracts, depending on the sign of the current, a magnetic field to the demagnetizing field in the magnet. This shifts the working point along the recoil curve of the material, see [44], which can change the magnetic flux density in the gap of the permanent magnet. By using a symmetrical design, two permanent magnets or two coils, this effect can be reduced. This effect is mostly proportional to *I*, since the recoil curve is very linear for rare earth magnets.

##### Change of the reluctance of the yoke.

The magnetic field produced by the coil adds to the magnetic field produced by the permanent magnet and can change the relative permeability of the yoke material, which depends in a non-linear function on the magnetic field, see [36, 37]. Consequently the reluctance of the yoke changes, causing a change in magnetic flux density in the air gap. This effect is, to first order, proportional to *I*^2^.

##### The reluctance force.

An iron core gets pulled inside a solenoid if it is energized, because in this position the magnetic energy of the system is minimal and so is the reluctance of the field path. The same is true for a coil inside a yoke. It experiences a force towards the point where the reluctance of the yoke completing the magnetic circuit of the coil is minimal [44]. This effect does not change *Bl*; instead it generates a force. This force is proportional to *I*^2^; hence, it would cancel if the currents were symmetric about zero. However, it is more complicated, because the coil moves between the mass on and mass off state due to the suspension’s finite spring constant. Hence, this effect can produce a different force on the coil, even if the current is absolutely symmetric.

##### Temperature change of the rare earth magnet.

The ohmic heating caused by the current passing through the coil heats up the magnetic material. With increasing temperature, the remanence of the material decreases causing a decrease of the magnetic flux density in the air gap. Sections 3.2.2 and 3.2.3 provide two ways of mitigating this effect: (1) by engineering a better magnet and (2) by actively heating the magnet in moving mode to keep the thermal load on the magnet constant during all modes of the Kibble balance experiment. This effect is proportional to the ohmic heating, i.e. *I*^2^.

##### Temperature change of the yoke material.

This effect is much smaller than the effect of changing the temperature of the active magnetic material, but it is listed for completeness. A changing temperature of the magnet system can change the reluctance of the yoke material and the geometry of the yoke, e.g. the width of the gap, through thermal expansion. Both effects change the magnetic flux density in the gap. This effect is also proportional to *I*^2^.

#### 3.2.2. Engineering of magnets with smaller temperature coefficients.

The preferred active magnetic material is samarium–cobalt, Sm_2_Co_17_, a rare earth magnet. A typical energy density of this sintered material is about 250 kJ m^−3^. Double this energy density is provided by neodymium-iron-boron magnets. However, for neodymium magnets the Curie temperature, the temperature where a magnetic material loses its magnetisation, is low, about 310 °C. Consequently, it has a large temperature coefficient of its remanence (of order 10^−3^ K^−1^). In contrast, samarium–cobalt has a Curie temperature of about 800 °C and its temperature coefficient is about a third of that of neodymium.

For a typical Sm_2_Co_17_ magnet the temperature coefficient of the flux density is about −3 × 10^−4^ K^−1^. If the magnet changes its temperature by −1 mK, the magnetic flux density changes by 3 × 10^−7^, a number that is about a 10 times larger than the relative uncertainty reached with Kibble balances. This is only acceptable, because the temperature drift is usually very slow compared to the cadence of taking data. By using an appropriate data sequence and data analysis, most of the drift can be rejected in the final result.

In recent years, magnet designs with much smaller temperature coefficients have been proposed. The data collected with these magnets will be quieter and it is less probable that the result includes a bias caused by temperature drift.

Researchers at METAS have designed a magnet system that uses gadolinium samarium cobalt [47] instead of samarium cobalt as the active magnetic material [42]. Alloying gadolinium to the samarium cobalt reduces the temperature coefficient from −3 × 10^−4^ K^−1^ to −1 × 10^−5^ K^−1^ at the expense of reducing the remanence by 30 %.

Besides using a better magnet alloy, another technique has been implemented in the METAS magnet system: temperature compensation with a shunt. This idea has previously been suggested by LNE [40]. The magnet system has a second return path for the magnetic flux. In the first path, the flux goes through the air gap, in the second path the flux goes through a magnetic shunt made from an iron nickel alloy with very low Curie temperature. Both flux paths are in parallel to each other. With rising temperature, the reluctance of the shunt path increases, which forces a larger fraction of the magnetic flux through the air gap. The thickness of the shunt can be finely tuned such that the increase in flux through the air gap is exactly equal and opposite to the loss of magnetization in the magnetic material. Hence, the magnetic flux density in the air gap remains constant, independent of the magnet temperature provided that no significant temperature gradients exist within the magnet. With this technique, it seems possible to build a magnet system with a relative temperature coefficient of the magnetic field in the gap within ±10^−6^ K^−1^.

#### 3.2.3. Actively controlling the temperature.

The relative change of the magnetic field inside the gap is the product of the temperature coefficient and the temperature change. The above section focused on minimizing the temperature coefficient. However, a similar end result can be achieved by reducing the temperature change of the magnet. A temperature change that is coherent with the sequence of the Kibble balance measurements is especially troublesome. Coherent temperature change can arise from several causes. For example, there is ohmic heating by the weighing current in the force mode, while there is none in the velocity mode. Hence, the heating power is modulated in phase with the experiment.

Researchers at NPL have implemented a simple but effective way to cancel this possible systematic effect [48]. The coil former carries a heater coil with the same resistance as that of the moving coil. During moving mode, a current equal to that of the weighing current is passed through the heater coil. The heater is a bifilar coil which does not generate an external magnetic field and therefore does not produce a force on the coil former or an external magnetic field.

Besides temperature changes due to power fluctuations inside the Kibble balance experiment, temperature changes from the environment (laboratory) can couple into the measurement, see section 3.11 for more details.

### 3.3. Voltage measurements

Voltage measurements are vital for the successful operation of both measurement phases of a Kibble balance. In the weighing phase it is necessary to measure the 5 mA to 20 mA current in the coil by measuring the voltage drop across a resistor. Usually the resistor is chosen to be between 200 Ω and 50 Ω to generate a voltage of order 1 V. In the moving phase most existing Kibble balances move the coil at approximately 1 mm · s^−1^. This speed is chosen to take a sufficient number of voltage measurements over a practical moving range of around ±20 mm. The voltage generated by the coil is usually chosen to be around 0.5 V.

In the moving phase, ground vibrations affect the apparent motion of the coil with respect to the magnet; this induces noise voltages in the coil [49, 50]. The interferometer system measures this motion and, in a well designed system, the voltage and velocity will be precisely correlated. If the measurements of both velocity and voltage are made with a bandwidth greater than that of the interfering ground vibrations, their ratio can be free of vibration generated noise. This condition sets a lower limit to the bandwidth of the input stage of the voltage measuring system of a few hundred hertz to 1 kHz. Usually the velocity and voltage signals are integrated over the same period and the ratio of average voltage to average velocity is determined by least squares data fitting. The joule balance uses the same technique of integrating the velocity and voltage signals over the same time but extends the integration time to points at which the coil is stationary.

For the weighing phase, there are no critical requirements for measurement bandwidth and voltage averages obtained by integration over a few seconds usually provide sufficient resolution.

#### 3.3.1. Measurement techniques.

A conventional voltmeter can measure voltages with an uncertainty of a few parts in 10^7^. To relate the measured voltages to fundamental constants with an uncertainty approaching 1 part in 10^9^, the measurements are usually made by connecting an accurate, Josephson-effect-based reference voltage in opposition to the majority of the voltage to be measured and measuring the difference, either directly with a digital voltmeter or, to reduce measurement noise, by a combination of a low noise amplifier and digital voltmeter. The voltmeter/amplifier is calibrated, at the expected values of the difference voltage using the voltage reference. By measuring differences in this way, the combination of voltmeter/reference can measure voltages which are stable at the 0.1%–1% level to a few parts in 10^9^.

#### 3.3.2. Josephson reference.

The Josephson effect [7] allows the construction of extremely precise voltage references by illuminating weak links between superconductors with microwave radiation of frequency *f*. The voltage generated is a multiple of *hf*/2*e*. In carefully fabricated and operated devices, the uncertainty of the voltage generated can be much better than 1 part in 10^9^. These references are commonly operated at frequencies of either 16 GHz or 75 GHz with associated voltages of 33 μV and 155 μV respectively. These voltages are too small for practical use at room temperature. However, arrays of these junctions, with output voltages up to 10 V, are available from several laboratories [51–54].

Two types of array have been used for Kibble balance work.

##### Hysteretic arrays

The hysteretic array uses insulating junctions and can be set to any multiple of *hf*/2*e* within its operating range [55]. However, such an array is very sensitive to electrical noise and interference which can cause it to suddenly change its voltage to a different (usually lower) multiple of *hf*/2*e* requiring extreme care in its use. Only one Kibble balance (the NPL Mark II [46]) has made direct measurements using such an array.

##### Programmable arrays

All existing Kibble balances either use, or intend to use, the programmable Josephson array [56–58] which consists of a string of Josephson junctions with a normal metal substituted for the insulator. Connections are made to the array to divide it into segments containing numbers of junctions that are usually related in a binary manner. Bias currents of ± a few milliamperes can be applied to individual segments to generate positive or negative voltages equal to the number of junctions in the segment multiplied by *hf*/2*e*. This allows the array to generate any voltage up to ± its maximum voltage in steps of *hf*/2*e*. Theoretically, a 75 GHz array can be set to match the voltage to be measured to within 155/2 ≈ 80μV requiring only a modest linearity and accuracy of the voltmeter measuring the difference.

The programmable array can change its voltage rapidly and this property can be used to simplify the voltage measurement procedure during the moving phase. A good approximation to the voltage generated by the coil can be calculated from the velocity of the coil and the magnetic flux density. This allows the array voltage to be continuously adjusted to null the input to the voltmeter during the acceleration and deceleration of the coil. This eliminates errors due to transient thermal EMFs generated by the switches which would otherwise be necessary to protect the voltmeter/amplifier from overload.

The output voltage of the array is not well defined during the time that it is switching from one voltage state to another so, to ensure the accuracy of the measured voltage, once the coil has reached its target speed [59], the voltage from the array is fixed and any changes are measured via the voltmeter/amplifier.

#### 3.3.3. Voltmeter.

As mentioned in section 3.3.2, in theory a voltmeter of fairly modest linearity and stability could be used to measure the difference voltages. In practice, the coil voltage noise is dominated by the effects of ground vibrations. In the weighing mode, the resistor voltage contains noise components from the servo system which keeps the balance in equilibrium. These noise sources limit, to around 1000, the gain of the amplifier which amplifies the difference voltage between the voltage source and voltage reference. Therefore the voltmeter must achieve part in 10^6^ level calibration, linearity and stability to achieve an overall measurement uncertainty of a part in 10^9^. Modern voltmeters can easily achieve this level of performance but often need time for carrying out internal ‘autozero’ procedures to eliminate the effects of drifts in their circuitry. This is not a major problem in the weighing phase of the measurement but does introduce problems in the moving phase where it is desirable to measure the voltage and velocity continuously over identical time intervals. The problem can be addressed by the NIST technique [60] which uses three voltmeters in a cyclic fashion where one is measuring, another is performing auto zero functions and the third is preparing to measure.

Another way of achieving the same end is to use a single voltmeter preceded by a highly stable preamplifier [48]. This can eliminate the need for autozeroing as the effective drift of the voltmeter is reduced by the gain of the preamplifier which becomes one part of the unavoidable linear temporal drifts in the measurement system. The reversals inherent in the measurement procedure allow such drifts to be removed from the measurement in the postprocessing calculations. The only problem with the use of a preamplifier is that most preamplifiers with nanovolt level drifts have low bandwidths which may be incompatible with the need to eliminate noise from the moving phase by correlation between the weighing and moving measurements. This problem is addressed in section 3.3.4.

#### 3.3.4. Amplifiers.

Most highly stable nanovolt level DC amplifiers [61, 62] are intended for use in measurements taking many seconds and have low bandwidths. Integrated circuit operational amplifiers can have much higher bandwidths but have unacceptable low frequency performance: they drift with time. By combining the two types of amplifier, it is possible to assemble a composite amplifier which has a bandwidth of several hundred Hz and nV level drift [48]. This can be achieved by using the low drift amplifier to monitor the difference in voltage between the inverting and non-inverting input pins of the high bandwidth amplifier. A simple servo loop feeding the offset nulling input of the high bandwidth amplifier drives the input voltage to zero ensuring that the composite amplifier is close to the performance of an ideal amplifier at low frequencies. Extreme care must be taken to minimise the effects of thermal EMF’s in critical parts of the circuit [48].

#### 3.3.5. Synchronisation of the voltmeter and counter.

To eliminate correlated noise between the average voltage and average velocity measurements in the moving phase, it is necessary for the signals to be integrated over the same time and for the bandwidths of both signal channels to be greater than the bandwidth of the noise signal. The velocity signal is a frequency, generated by the laser interferometer, which is measured using a frequency counter. By using passive noise isolation techniques, such as resilient pads in parts of the support structure of the balance, it is possible to limit the bandwidth of vibrational noise to a few hundred hertz. Under these circumstances, differences in the integration times of less than 1 ms will have little effect on the elimination of correlated noise. Many methods of achieving this aim are possible and indeed have been implemented. For illustration, we will describe a method similar to the one used on the NPL Mark II balance [48] but employing a different form of frequency counter. It provides simple and accurate measurements of velocity and voltage with the caveat that the integration times are not identical.

The technique uses a charge balance voltmeter and a time interval analyser. Both of these instruments possess the great advantage that they measure continuously. The charge balance voltmeter can be considered to be an integrator with two inputs. The first is connected directly to the voltage to be measured, the second is connected alternately to positive and negative reference voltages. The reference input polarity is switched whenever the output of the integrator reaches one of two fixed limits. The times for which each reference is applied are accumulated and, upon receipt of a trigger pulse, the analogue-to-digital converter (ADC) waits until a whole number of reference switching cycles has taken place and reports the accumulated data. The average value of the input voltage can be calculated from this data and calibration information. The period of the reference switching cycle is chosen to be around 200 μs and varies about this value.

The time interval analyser counts events and notes the time of the first and last event. This allows the average frequency of the events to be calculated but, as the last event of one measurement corresponds to the first event of the next, the counter, like the voltmeter described above, measures its input continuously. Whenever it notes a time it generates an output pulse. This ‘gate pulse’ is used to trigger the voltmeter which will take approximately 200 μs to respond so, on average, the times of the integrals will be shifted by 100 μs with a jitter of ±200μs. This may cause a slight decrease in the correlation between voltage and velocity signals but both integrals will be correct. There is a further advantage to the back-to-back data collection in that no part of either signal is lost and, if desired, the integration time can be increased in post processing, giving the ability to analyse and correlate the data at both higher and lower frequencies.

#### 3.3.6. Joule balance measurements.

In its equivalent to the Kibble balance moving mode, the joule balance adopts a similar measurement scheme to the Kibble balance except that the integrals mentioned above are taken between points where the balance is stationary. This reduces the velocity integral to a simple difference in position of the coil. However, increased care is required in the measurement of the coil voltage integral. As the motion of the coil must be started and stopped the reference voltage must be varied to keep the voltmeter in its linear range. A programmable Josephson voltage reference does not produce well defined transitions between voltages (section 3.3.2) and this can increase the uncertainty of the measurement. This problem is solvable and is presently under investigation [63, 64].

### 3.4. Current generation and measurement

**Figure 4.**
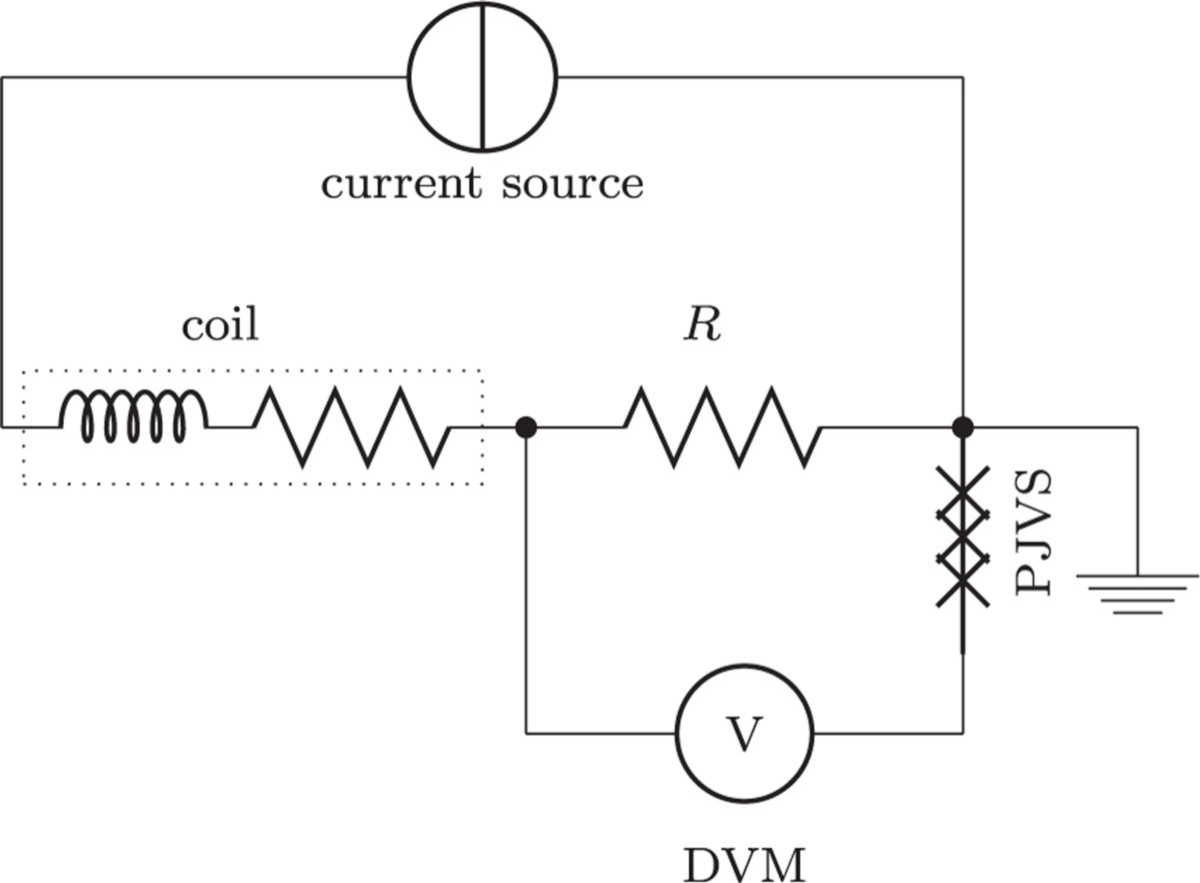
Typical circuit topology during force mode. The current is passed through the coil, drawn as an inductive and a resistive element and a measurement resistor. The voltage drop across the measurement resistor is compensated with a programmable Josephson voltage system (PJVS) and the residual voltage is measured with a digital voltmeter (DVM).

In weighing mode, a current is passed through the coil to generate the electromagnetic force. The current continues on through a resistor and the potential difference across this resistor is measured precisely, in a four terminal geometry, using the techniques described in section 3.3. Figure 4 shows a simplified circuit diagram of a Kibble balance in weighing mode. Typically, the electromagnetic force is half the weight of the mass standard used in the experiment and the current is reversed, such that the difference of the two electromagnetic forces corresponds to the weight of the mass. In this section, we discuss the current sources, the measurement resistor, and the process required to calibrate the measurement resistor using the quantum Hall effect.

#### 3.4.1. Current sources.

Typical weighing currents range from a few milliamperes to about 20 mA, given by the quotient of the geometric factor (see column 6 of table 1) and half the weight of the test mass. The current source needs to be able to generate a bi-directional current, since the force reverses between the mass-on and mass-off measurement. Properties to consider designing a current source are: (1) the impedance to ground, (2) the update rate, (3) the resolution of the digital to analogue converter (DAC), and (4) the noise of the current source. Details to each of these design considerations are discussed below. Descriptions of current sources for Kibble balances can be found in [48, 65].

The current source should be fully isolated to allow the experimenter to choose the point at which the Kibble balance measurement circuit will be connected to the mains ground wire. If the circuit is connected to mains ground at more than one point parasitic currents can flow which can introduce a bias to the experiment if they flow through the coil or resistor, but not both. For example, a current flowing through the coil but not through the resistor, will contribute to the electromagnetic force, but it will not be measured, resulting in a bias in the experiment. In order to avoid such parasitic currents, all elements of the electrical measurement circuit should have a high resistance to ground. High in this context is approximated by dividing the resistance in the measurement circuit by the maximum acceptable bias. For example, the current measurement requires a relative uncertainty of 1 part in 10^9^ and a 100 Ω resistor, the resistance to ground should be more than 100 GΩ and would usually be at least 1 TΩ. One possible path of parasitic resistance is via the cables that carry the control signals from the control computer to the current source. A good way to minimize this leakage path is to employ fibre optical communications between the controller and the current source, e.g. see [66]. Another leakage path is via the power supply for the circuit. This leakage can be eliminated either by powering the current source with batteries or by using a mains power supply which has been carefully isolated. Such a supply is described in [67]. This is a general problem for the Kibble balance and is further addressed in section 3.11.4.

As a rule of thumb, the update rate of the current source should be at least an order of magnitude faster than the closed loop bandwidth. This reasoning gives a lower bound for the update rate of the current source. The faster the update rate of the current source the better. However, the closed loop system in the weighing mode contains at least two low pass filters, attenuating the effect of changes at the current setpoints at high frequencies. The resonance frequency of the mechanical system of the Kibble balance depends on the design but is in the ball park of tenths of Hz to tens of Hz. This leads to an attenuation of the quotient of balance position and coil current at high frequencies. The second low pass filter is electrical and is given by the inverse of the time constant of the system composed of the self inductance of the coil and the series resistance of the measurement resistor and the coil, i.e. *τ* = *L*/*R*. For example, a self inductance of 1 H and a total resistance of 100 Ω yield a time constant of 10 ms. A Kibble balance coil in a permanent magnet has typically an inductance of a few henries. The total resistance is typically below 1 kΩ. Hence, this low pass filter attenuates the quotient of coil current to applied voltage for frequencies above 1 kHz. This effect can be modified by the internal feedback of the current source.

The simplest design of the current source is a variable voltage source followed by a transconductance amplifier. In this case, the resolution of the current source is given by the resolution of the voltage source. Naively, one would think that in order to measure the current with a relative uncertainty of 10^−9^, a resolution of 10^−9^ is required. However, this requirement calls for a digital-to-analogue converter (DAC) with 30 bit resolution, which is not commercially available. One technique to obtain sufficient resolution is to add two voltages together with a summing amplifier, where one voltage is attenuated by a voltage divider. In the NPL balance [48], for example, two 16-bit DAC outputs are combined with a relative gain of −2000 : 1. In the NIST Kibble balance [65], two 20 bit DAC outputs are combined with a ratio of 1000 : 1. The ADC with the larger gain is used for coarse control of the balance and the one with the smaller gain for fine control. Since the current source is in a closed loop feedback system, a higher resolution than the nominal resolution will be achieved, because the Kibble balance will average or integrate the applied current with a time constant given by the differential equation of the balance. Hence the output rate multiplied with this characteristic time gives an effective increase in resolution of the DAC.

The combination of the two DACs will not be linear to their combined resolution but, if the control software is written to minimise unnecessary changes in the output of the more significant DAC, the resolution will, for most of the time, be equal to that of the less significant DAC. When it is necessary to change the more significant DAC, it is set to centre the less significant DAC in its range; this increases the time to the next change and its associated small glitch in the combined output.

The noise of the current source is one contribution to the measurement noise in the weighing mode and ultimately the type A uncertainty. However, this part is not likely to be the dominating factor in the measurement noise. In this context, it is best to think about the current noise in the frequency domain. Depending on the frequency, the noise level of the current source varies, achieving worst levels at low frequencies due to 1/*f* noise. However, this is not a problem. The critical time scale is given by the bandwidth of the weighing servo. In weighing mode, the servo adjusts to keep the balance position constant over many minutes without applying rapid changes of current which would be seen by the measurement system as noise. In practice, the bandwidth of the servo is of the order of 1 Hz. The action of the servo eliminates the need for excessive low frequency stability in the current source. For some balances, e.g. METAS, BIPM, a fixed current is required and such circumstances often require a highly stable current source. However, care should be taken to minimise the noise of the current source at frequencies higher than the bandwidth of the servo. As the weighing data is usually taken by a series of averages, each lasting a few seconds, the sensitivity of the integral used to form the average, to high frequency noise drops linearly with the frequency of the noise. This indicates that close attention should be paid to the noise of the source in the region around 1 Hz to 100 Hz.

In a practical situation, the slow drifts in the balance due to outgassing and temperature changes usually dominate over random noise. If the dynamic range of the less significant DAC is chosen to ensure that it is many times the change expected over the duration of a single weighing, the weighing current should be glitch free during each measurement.

To further reduce the mid-range noise it is possible, once the balance has stabilised, to reduce the bandwidth of the more significant DAC. As it, and its reference, will contribute the majority of the current source noise, a significant reduction in its bandwidth should result in a significant reduction in the critical mid-range noise.

#### 3.4.2. The measurement resistor.

The measurement resistor in the Kibble balance is, typically, a conventional resistor (wire wound or thin film). This conventional resistor is calibrated against a quantum Hall resistor (QHR) on a regular basis (see next section). It is used in a four terminal configuration: two terminals connect to the current leads and two connect to the potential leads. The potential leads are connected to a voltmeter with high input impedance. Ideally, no current is flowing in the potential lead, hence contact resistances in the potential leads do not bias the measurement.

The measurement resistor is kept either in an air or an oil bath with good temperature stabilization. Nevertheless, the temperature of the bath must be carefully monitored. Most resistors have a linear temperature coefficient of several μΩ Ω^−1^ K^−1^. For certain resistors, in addition to the linear temperature coefficient, a quadratic temperature coefficient must be considered. Besides the temperature dependence, the resistance depends on the measurement current, mostly due to self-heating. A power coefficient, i.e. the change in relative resistance divided by the power dissipated in the resistor, is used to quantify this effect. If the calibration current is different from the current used in the Kibble balance, a power correction may become necessary. Some resistors, with a certain design, require an additional pressure correction which includes changes in the atmospheric pressure and the hydrostatic pressure, exerted by the oil above the resistive element. For these resistors, the ambient atmospheric pressure must also be monitored.

In some cases, the resistor can cause local heating of the oil surrounding it which can cause transient effects at the start of the weighing mode. If the element is surrounded by a can, the removal of the can may allow more efficient mixing of the oil which may reduce the heating effect to acceptable levels. Otherwise it may be possible to use isolated heaters, near the resistance element, to keep the power dissipation in the oil bath constant.

#### 3.4.3. Resistance determination with the quantum Hall effect.

To link mass to the Planck constant the resistor used in the Kibble balance measurement must be related to the quantum Hall effect (QHE). A QHE measurement system consists of a superconducting magnet, a QHE device and a cryogenic current comparator bridge. The QHE device is held in a low temperature probe in the 5 T to 14 T field of the superconducting magnet. The Hall resistance of the quantised Hall sample is compared to that of the Kibble balance resistor using a cryogenic current comparator [68, 69]. The voltages at the potential terminals of the two resistors are adjusted to be equal by passing different currents through them and the ratio of the resulting currents is measured using a technique which makes use of the Meissner effect. By this means, the Kibble balance resistor can be measured with an uncertainty of a few parts in 10^9^.

As mentioned above the value of the resistor will depend on the power dissipated in it. If possible, the resistor should be calibrated at the currents that are used in the Kibble balance measurements. If this is not possible, a power coefficient must be measured which can then be used to correct the value of the resistor to the operating power. The use of a power coefficient will, in general, increase the uncertainty associated with the resistor.

Ideally, the resistor should be measured *in situ*, either by using cables [48] over tens of metres, or via a transportable QHR measurement system [70]. If it is necessary to transport the resistor to the quantum Hall effect system, extreme care should be taken in the transport arrangements. Mechanical shocks can alter the value of the resistor in an unpredictable way and would increase the uncertainty assigned to the resistance measurement. Extremely good temperature control and monitoring is also required. A thermal shock to the resistor, caused by changing thermal environments during transport, could permanently change its value or its temporal drift. In addition, if the temperature gradients in the enclosure varied from the location of use to the location of measurement, and this affected the temperature difference between the monitoring thermometer and the resistor, the resistor value, corrected for temperature, would be different at the two locations. The magnitude of this problem can be investigated by changing the temperature gradient across the resistor enclosure at a constant temperature as seen by the monitoring thermometer. There should be no significant correlated changes in the device resistance.

### 3.5. Velocity and position measurement

#### 3.5.1. Interferometry.

Laser interferometry is used to relate the vertical velocity of the coil/mass pan to the metre and the second and to monitor the position of the coil/pan. In some Kibble balances, additional interferometers are used to monitor the rotation of the coil.

#### 3.5.2. Refractive index of air.

The wavelength of light in air *λ*_air_ is altered from its vacuum value *λ*_vac_ such that *λ*_vac_ = *nλ*_air_ where *n* is the refractive index of air. If a Kibble balance is operated in air, a correction must be made for the refractive index of the air, which depends upon its temperature, pressure and composition and is of the order of 300 parts in 10^6^. However, most existing Kibble balances are operated in vacuum, at a pressure of less than 0.1 Pa, which makes the this correction and the correction for the effects of air buoyancy on the working mass (section 3.7.5) negligible.

#### 3.5.3. Light source.

All existing Kibble balances use visible lasers to provide the length reference for interferometry. The frequency of the light emitted by the laser needs to be stabilised to achieve the uncertainties required. An excellent stabilisation method involves locking the laser frequency to a line in the spectrum of molecular iodine [71]. Such iodine-stabilised lasers can produce almost monochromatic radiation with frequency stabilities far better than 1 part in 10^9^ and, as many of the lines are well characterised and recommended for the practical realisation of the metre [72], such a laser can act as a primary standard of length. Alternative schemes, such as Zeeman stabilisation [73] are commonly used but require calibration against a primary standard in intervals of months.

The light from the laser can be coupled into the interferometer either by free space propagation or by use of an optical fibre. Free space propagation has the advantage that the verticality of the beam can be checked and adjusted from outside the vacuum chamber which houses the Kibble balance, but has the disadvantage that the laser housing has to be maintained in alignment with the apparatus within the chamber, a requirement which is often difficult and time consuming to achieve. Fibre coupling frees the laser to be placed anywhere in the laboratory and, if the fibre is passed through a vacuum seal into the chamber, avoids the need for optical windows in the vacuum chamber wall. However, this requires that a procedure be established to ensure that the laser beam is vertical within the chamber.

#### 3.5.4. Types of interferometer.

So far, two different types of interferometers have been used in Kibble balances, Michelson and Fabry–Perot interferometers. Table 2 shows the types of interferometer used in Kibble balances at different laboratories.

##### Michelson

In a Michelson interferometer, the light is split into two arms, the measurement arm and the reference arm. The length of each arm is the distance from the beam splitter to a reflector. The reflector of the measurement arm is mounted on the moving coil. The measured optical distance is twice the displacement of the reflector in the measurement arm. This ratio of optical distance to actual displacement can be increased using multiple passes. Based on the frequency difference of the light entering the two arms, a distinction between an homodyne and heterodyne interferometer is made.

##### Homodyne

In a homodyne interferometer, the optical frequency of the light entering both arms is identical. Hence, if the lengths of both measurement arms remain constant, the interference signal at the output port has a constant brightness. Moving the measurement reflector will cause the output port’s brightness to go through fringes, i.e. change from dark to bright to dark. The period of this signal change corresponds to a change in the optical path length by one wavelength. A Michelson type interferometer has two output ports, referred to as dark port and bright port. In a conventional beamsplitter energy conservation requires the sum of the luminous fluxes to add to a constant, if one port is bright the other is dark and vice versa. However, the beamsplitter used in the NPL/NRC interferometer is deliberately made lossy to allow the determination of the position of the coil at low velocity [74]. Monitoring both ports allows rejection of the intensity fluctuation of the laser and the subdivision of the fringes. If the measurement reflector moves with constant velocity the fringe crossing frequency is given by the 2*Nv*/*λ*, where *v* is the velocity of the coil and *λ* the wavelength of the laser and *N* the number of passes.

##### Heterodyne

In a heterodyne interferometer [75], light enters the two arms with frequencies that differ by a modulation frequency ranging from about 1 MHz to 50 MHz. If the reflector of the measurement arm is stationary, the interference signal is at this frequency difference. If the reflector moves with a velocity *v* along the line of sight of the measurement beam, the interference signal is Doppler shifted by 2*Nv*/*λ*. The sign of the frequency shift encodes the direction of motion.

The essential difference between a homodyne and a heterodyne interferometer is the detection frequency, which is increased by the modulation frequency in the heterodyne interferometer. This is one point worth considering when deciding between using a homodyne or an heterodyne interferometer. The other important consideration is the optical non-linearity [76]. In brief, optical non-linearity occurs when light that is assumed to be in the measurement arm leaks into the reference arm and vice versa. In the homodyne case, the incident laser beam is, sometimes, separated by the polarization state into the measurement and reference arm and, hence, the optical non-linearity is caused by polarization mixing. Polarization mixing occurs if the polarizing beam splitter is not perfect. Part of the light that should have been reflected is transmitted instead. Similarly, for a heterodyne interferometer, light of the wrong frequency can leak into the other arm.

This effect is called frequency mixing. Another mechanism for frequency mixing to occur, is when the two polarization directions have different frequencies and some of the light not intended for part or all of the interferometer leaks into these areas due to imperfections in the optical elements. Both optical non-linearities, frequency and polarization mixing produce biases that are periodic in displacement. The period is referred to as fringing period. By averaging the data over a fringing period, this error can be attenuated. The optical non-linearity can be a limiting factor in the achievable signal-to-noise ratio of the velocity mode measurement.

##### Fabry–Perot

A Fabry–Perot interferometer requires only one arm. Two mirrors in the arm are aligned such that they form an optical resonator, commonly called a cavity. The reflection coefficient of the cavity changes periodically with a period of *λ*/2. A photo diode measures the light reflected from the cavity. Similar to the homodyne detection the velocity signal produces a fringing frequency of 2*v*/*λ*. So far, only one National Metrology Institute, METAS, is using a Fabry–Perot interferometer [21, 77].

The current Kibble balances that use Michelson interferometers employ corner cubes as reflectors for the measurement arm. The METAS Kibble balances use flat mirrors to form the Fabry–Perot cavity. Flat mirrors are much smaller than corner cubes, an important factor if the mirror has to be mounted inside the narrow air gap of a permanent magnet.

Systematic biases in the interferometric velocity measurement can arise from restricted width of the beam and distortions of the wavefronts and angles between the reference beam and the measurement beam. The wavefront distortions are especially troublesome if the moving reflector is subject to parasitic motions, e.g., perpendicular to the measurement direction, that may change the wavefront. A comprehensive description of these biases can be found in [13, 78–80].

#### 3.5.5. Alignment of the laser beam to the vertical.

Correct operation of a Kibble balance requires that only the vertical velocity of the coil/mass pan is measured. This requires that the interferometer’s laser beam is accurately vertical at the point where it reflects from the reflector which is used to measure the velocity. A simple way to achieve this is to allow the laser to reflect from the surface of a pool of liquid inserted into the beam path. The centre of the surface of an undisturbed liquid pool is horizontal. If the angle of the laser beam is adjusted so that the incoming and outgoing beams are exactly coaxial, the beams will be normal to the surface and therefore vertical. A simple way to detect coincidence of the beams is to observe the reflected beam when it hits the periphery of the entrance pinhole for the laser beam. The angle of the beam is adjusted until the reflected beam exits through the pinhole. The adjustment can be made with a resolution of about 0.1 mm. If the pinhole and reflector are separated by about 2 m, the beam can be aligned to the vertical to about 25 μrad. If the beam is not vertical a cosine error results. Using the expansion cos(*α*) ≈ 1 −*α*^2^/2, the term *α*^2^/2 must be below 1 part in 10^9^. This requires that *α* is less than 45 μrad. The simple technique described can fulfil this requirement. The verticality requirement described above is relatively easy to achieve, since the measurement bias is proportional to the angle squared. Another bias, can occur, when the coil has a parasitic horizontal velocity, for example, *u_y_*. In this case, if the laser beam deviates from vertical in the direction of the *y* axis by *α_x_*, a measurement bias of *α_x_u_y_* occurs [81].

A number of techniques have been used for this adjustment; some substitute a tiltmeter and mirror for the liquid surface to avoid problems with handling liquids [5]. If the laser beam is introduced into the vacuum chamber from outside and is not deviated before it is reflected from the coil or mass support, the verticality of the beam can be determined externally. If, however, the laser is introduced via a fibre into the chamber laser verticality must be measured [82] and adjusted in vacuum or it must be ensured that evacuating the chamber does not affect the verticality of the laser beam. Motorised mirrors can easily be used to adjust the beam angles. To measure laser vertical, either of the general techniques described above may be used but care must be taken to avoid liquid evaporation in the vacuum chamber or changes in the sensitivity and offset of a tiltmeter on transition from vacuum to air. A possible solution to using a liquid mirror open to vacuum without evaporation problems was suggested in [83] using gallium which when gently heated forms a liquid mirror with very low vapour pressure.

#### 3.5.6. The Abbe error.

The laser beam retroreflector is always mounted so that, in moving mode, its angular velocities *ω_x_* and *ω_y_* about the horizontal axes *x* and *y* are minimised. If the effective point of measurement of the velocity *u* is not on a vertical line through the centre of mass but is offset from it by distances *r_x_* and *r_y_* the angular velocity will be coupled into the measurement of vertical velocity. For small angles, the measured velocity *u_m_* can be approximated to be:

(25) $$u_{m}=u-r_{x}\omega_{y}+r_{y}\omega_{x}.$$

To achieve an overall contribution to the uncertainty of the measurement of a part in 10^9^ for angular velocities of 0.1 μrad s^−1^ and a coil velocity of 1 mm s^−1^, *r_x_* and *r_y_* must each be less than 5 μm.

In an apparatus which uses a single interferometer, the adjustment can be made by fixing the balance beam and causing the arm of the balance containing the retroreflector to perform pendulum oscillations. The horizontal position of either the centre of mass or the retroreflector can then be adjusted until there is no apparent vertical motion, as seen by the interferometer, at the fundamental frequency of the pendulum motion.

For balances which use three interferometers placed around the periphery of the coil, the adjustment process is carried out mathematically by weighting the contributions of the three interferometers to the calculation of the velocity of the coil [84].

#### 3.5.7. Synchronisation of velocity and voltage measurements.

To reduce the noise of the moving measurement, the measurements of vertical velocity must be synchronised to those of the voltage generated by the coil. The techniques for achieving this are discussed in section 3.3.5.

#### 3.5.8. Provision of a time reference.

The Kibble balance requires a reliable and accurate time reference which is traceable to the SI. As this reference is needed at an uncertainty better than a part in 10^9^ and the primary clocks which maintain the SI unit of time operate at levels better than parts in 10^16^ this is not usually a problem [85].

Many NMIs have one or more Hydrogen Masers which are referenced to primary clocks. The output of the maser is usually distributed as a 10 MHz signal via lab-wide optical fibres and coaxial cables. In addition, modern GPS-disciplined oscillators, which take their long-term time reference from visible GPS satellites, can be used, provided that the oscillator which is controlled has excellent phase noise and sufficient medium term stability to work properly when few satellites are available [86, 87]. Rubidium oscillators can also be considered but their drift can be affected by helium in the atmosphere and they must be checked periodically against a primary standard. Caesium clocks which are used to realize the definition of the second can be used directly. Such signals usually drive the reference input of the critical counters/synthesisers in the system (those for the velocity measurement and the frequency reference for the microwave synthesiser which drives the Josephson array).

In all cases, it is useful to have an indication of the correct operation of the standard and this is routinely provided by high-quality GPS-disciplined oscillators. It is also prudent to have a secondary mechanism for checking the frequency references via an independent route to a primary standard. A possibility for this is to use an off-air frequency standard, which makes use of a low frequency radio signal whose carrier is locked to a primary standard. In general such standards do not have the low phase noise required for direct use but, by using measurement times of more than 1000 s, can provide a valuable consistency check at relatively low-cost.

### 3.6. The local acceleration due to gravity

To derive the mass *M* from the weight *Mg*, which is measured by the Kibble balance, it is necessary to know the value of the acceleration due to gravity *g* at the centre of gravity of the mass during the weighing phase of the measurement [88, 89]. A number of geophysical effects cause the value of *g* to vary with time and position. In general, *g* is measured at a different place and time than those required; therefore, the measured value must be corrected to compensate for this.

An absolute gravimeter is necessary to measure *g* with an uncertainty of a few parts in 10^9^ which is necessary for a low uncertainty in the overall measurement. Absolute gravimeters are expensive instruments which are also time consuming to set up and operate. Thus a number of procedures exist for combining the measurements made by absolute gravimeters with those made by the Kibble balance; this section will discuss their relative merits.

#### 3.6.1. Absolute gravimeters.

Absolute gravimeters operate by dropping a mass in a vacuum and timing its fall. The dropped mass can be either macroscopic [90] or atomic [91, 92]. At present, the most common gravimeter used with Kibble balances drops a macroscopic reflector. The reflector is incorporated into an interferometer and the passage of interferometer fringes caused by the fall of the object in vacuum are timed. If the laser frequency and the time reference are calibrated in SI units, the instrument measures *g* in SI units with an uncertainty of a few parts in 10^9^.

#### 3.6.2. Relative gravimeters.

As their name implies, relative gravimeters [93–95] measure relative changes in *g*. They are used for two main tasks: gravitational surveys to support the transfer of the value of *g* from the gravimeter to the Kibble balance and measuring changes in *g* with time to support less frequent absolute measurements of *g*.

Many relative gravimeters measure changes in length of carefully designed spring systems [96]. These instruments are small, easy to move, easy to operate and are usually used for three dimensional gravity surveys of Kibble balance laboratories.

Superconducting relative gravimeters sense the movement of a magnetically levitated niobium sphere and are costly, extremely sensitive, highly reliable, but difficult to move [97]. They do have the advantages of relatively low maintenance and, once set up, they are very easy to operate. These instruments are useful to support the temporal interpolation of *g* between measurements made with absolute instruments.

#### 3.6.3. Methods of operation.

The ideal way of measuring *g* for use with a Kibble balance is to operate an absolute gravimeter simultaneously with the weighing phase of the measurement. Under these circumstances, the only corrections needed are those which transfer the position of the measurement, a correction for the speed of light, which is required by the gravimeter, and possible corrections for the finite masses of the gravimeter and watt balance. Whilst this method gives good results, it increases the effective complexity of the system and thereby decreases its reliability and increases its cost to operate.

If a superconducting relative gravimeter is available, it can be used to interpolate between infrequent absolute measurements again needing only the same corrections as mentioned above. If an interpolation instrument is not available and the absolute instrument is only available infrequently, it is necessary to use the average value of *g* from the gravimeter measurements corrected for the geophysical effects listed in section 3.6.5. The Kibble balance software calculates the instantaneous value of *g* at the required time from estimates of these effects. On a well characterised, properly instrumented, stable site, this technique can yield a relatively modest added uncertainty to that of the measurements of the average value of *g*.

#### 3.6.4. Gravity surveys.

Before a Kibble balance is constructed on a new site, it is important to carry out a gravitational survey. A minimal survey will determine the vertical and horizontal gravitational gradients at the planned locations of the absolute gravimeter and the Kibble balance mass pan and the horizontal and vertical transfer corrections between these points. A more thorough survey will provide a 3-dimensional map of the site from which the optimum locations of the Kibble balance and gravimeter can be determined [95, 98–100]. These are usually at maxima or minima of the field as a function of horizontal position so that the corrections are insensitive to small positioning errors in either the Kibble balance or the gravimeter.

#### 3.6.5. Corrections.

A number of corrections must be considered when deriving an accurate value of *g* at the centre of gravity of the mass from a set of raw measurements of *g* made by an absolute gravimeter.

##### Speed of light correction

This correction is applied in the gravimeter software and reflects the fact that, at the required uncertainty of the measurement, the speed of light cannot be considered to be infinite with respect to the velocity of the falling object.

##### Horizontal correction

This correction is determined by the survey discussed in section 3.6.4 and should be stable unless significant masses have been moved in the vicinity of the gravimeter or Kibble balance. Ideally it should be zero but in practice, due to the location of masses such as room walls, it is often a few parts in 10^9^.

##### Vertical correction

Part of this correction arises from gradients measured during the survey discussed in section 3.6.4. It is useful to design the Kibble balance so that the height of the mass pan is close to part of the drop of the gravimeter which reduces the height difference, the size of the vertical correction and thereby its uncertainty. The final correction will depend on the value determined from the survey, any change to the height of the reference plane of the mass pan and the height of the centre of gravity of the mass above the reference plane of the mass pan.

##### Atmospheric pressure

The measured value of *g* will be decreased if the mass of the part of the atmosphere above the apparatus increases. This is reflected in an increase in the local barometric pressure. Usually a single coefficient is used to calculate the correction to *g* from the measured barometric pressure. This assumes that the pressure in the region a few kilometres around the Kibble balance is uniform which is usually reasonable except under storm conditions. Some modern laboratories have air conditioning systems which raise the pressure inside the laboratory. Under these circumstances, it is necessary to ensure that the barometer used for the measurements is recording the outside air pressure.

##### Solid earth tide corrections

Changes in the relative positions of the sun and the moon have a significant effect on *g*. These effects are known as the solid earth tides and are modelled by considering the earth as a solid body which is not deformed by the gravitational forces acting on it. The geophysical community has carried out considerable work on the modelling of the solid earth tides and they can be predicted accurately, given input parameters of the time and the location of the Kibble balance on the surface of the earth. Real-time corrections can be made either by incorporating solid earth tide prediction code into the program controlling the Kibble balance or by interpolating the correction from a table produced by a stand-alone tidal prediction program. If real-time tidal correction is not required, the same techniques can be used in the post processing of the results.

##### Ocean loading corrections

The earth is an elastic body and further corrections can be made to take this into account. As its name implies, the principal source of the correction is the change in height caused by the tidal motion of sea water. The correction depends on the location of the Kibble balance with respect to large bodies of tidal water. In many cases the correction is small and can be ignored.

##### Earth rotational axis (polar motion) correction

The rotation of the Earth about its axis provides an acceleration of the laboratory frame of reference which affects the measured value of *g*. If the Earth rotated about a fixed axis, the effect would be constant. Unfortunately the point at which the instantaneous rotational axis of the Earth intersects the surface of the earth moves very slowly in a spiral pattern. The location of this point is monitored and its location is published on line by the international earth rotation and reference systems service (IERS) from which a correction can be calculated. This effect is also referred to as polar motion.

##### Self-mass corrections

Both the gravimeter and the Kibble balance contain parts which have significant masses. For a typical gravimeter, the associated correction [101, 102] of *g* is a few parts in 10^9^ but for a Kibble balance the correction associated with the magnet can easily be 20 parts in 10^9^ and other parts of the apparatus will have smaller effects. There are presently two ways to achieve a low uncertainty on this correction. A finite element model can be used to calculate the gravitational field and its gradient near the mass pan to allow the appropriate correction to be applied at the centre of gravity of the working mass. Alternatively, a relative gravimeter can be placed in the area usually occupied by the mass pan to measure the difference in *g* from a local reference point and the vertical gradient near the pan. This technique depends on having a sufficiently low magnetic field in the vicinity of the mass pan to operate the gravimeter and a large enough space to accommodate the instrument.

#### 3.6.6. Verification of the correct operation of an absolute gravimeter.

An absolute gravimeter is a complex instrument and, whilst care in its calibration and operation should achieve low measurement uncertainties, the measurement results will vary naturally because of local and global geophysical effects. This makes it desirable to verify the operation of the instrument and this is usually achieved by comparison with other similar instruments in gravimeter comparisons [103, 104]. Participation in such comparisons is relatively expensive as the gravimeter must be shipped to the site of the comparison and operated there. However, successful participation in a comparison provides independent evidence that the instrument, which is a critical part of a Kibble balance, is both operating correctly and has a valid uncertainty budget.

### 3.7. Measurement of the working masses

Most conventional mass metrology is performed in air. If the masses being compared have exactly the same density, the effect of buoyancy due to the mass of air displaced by the mass will be equal for both masses and will cancel. If a Kibble balance is operated in air, the apparent weight of the mass is compared to the force generated by the coil. Under these circumstances, the effects of air buoyancy do not cancel and the results must be corrected for its full effect which is approximately 500 parts in 10^6^ for a silicon mass. To achieve their target uncertainties at a few parts in 10^8^, the Kibble balances have to operate in vacuum to eliminate the significant uncertainty arising from the air buoyancy corrections. This means that the measurements made in vacuum must be related to those made in air and this can be done without the need for buoyancy corrections [105]. On moving a mass from air to vacuum, layers of molecules (mostly water) present on the surface are removed and the desorbed mass must be taken into account [11]. Considerable work has been carried out in this area [106–108] and different techniques [109] exist to estimate the mass change due to the removal of the sorption layer. In an air-vacuum comparator, the test mass can be compared to a sorption artefact which has the same mass, identical surface properties but a known surface area several times that of the test mass [110]. Differential changes in mass between the two artefacts when moved between vacuum and air can be used to measure the mass lost or gained per unit surface area of the mass.

Another, more direct, way to compare a mass in vacuum to a mass in air is via a mass comparator that has two mass pans, one in vacuum and one in air that are connected via a magnetic coupler. Such a magnetic suspension mass comparator (MSMC) has been built at NIST [111]. It will be interesting to see what uncertainties can be achieved with such an MSMC.

The process of disseminating the unit of mass from a unit of mass realized in vacuum is described in other articles in this focus issue of *Metrologia*.

#### 3.7.1. Substitution weighing.

In the watt balance, the working mass is measured by substitution weighing. For reasons described in section 3.2, the balance is offset by half the weight of the working mass. This requires a current to flow in the coil to generate an upwards force of half the weight of the working mass. This current is measured and the mass is lowered requiring the current to be reversed to maintain the balance in equilibrium. The weight of the mass is derived from the difference in the two currents and the reversal makes the measurement insensitive to constant thermal EMF’s in the measurement circuit. If the balance is not disturbed by the raising or lowering of the mass, the technique can make extremely accurate mass measurements.

#### 3.7.2. Types of balance.

A range of balance types are used in Kibble balances their principal features are summarised in table 3.

NIST and NPL/NRC balances use knife edges which are robust and allow the beam/wheel to rotate enough to move the coil in the moving phase; however, they suffer from hysteretic effects [48, 115] which must be eliminated by moving the beam/wheel in a damped sinusoidal manner. This sinusoidal motion is executed after every mass transfer to the mass pan. The sinusoidal motion wastes time and can increase the weighing noise.

MSL will use a pressure balance for weighing. The piston of the balance provides precise vertical motion during the moving phase.

The majority of Kibble balances use mass comparators which are sensitive balances which use flexures, i.e. thin metal strips which are used as highly repeatable pivots. The flexures provide high sensitivity and low hysteresis but to avoid damaging them, their motion must be limited, so a separate mechanism must be provided for the moving phase.

#### 3.7.3. Alignment of the mass on the mass pan.

For a beam balance, the mass pan is suspended at the end of the beam by a flexure or a knife edge. If the mass is not centred on the mass pan, a torque will be applied to this pivot. Due to the finite stiffness of real pivots, a fraction of this torque is transmitted to the beam. This parasitic torque can cause a measurement bias, referred to as corner load error. This error can be reduced by implementing multiple pivot points between the mass pan and the beam. With each pivot point the amount of torque that is transmitted up the linkage is substantially lowered.

The mass pan can be designed such that the mass is self centring [116, 117]. Then the mass ‘walks’ to the centre of the balance pan with each mass exchange. A couple of weighings using such a design will reduce the corner loading effect.

A pendulum motion of the mass pan can increase the noise of the watt balance, substantially increasing the number of cycles required for the mass self-centring action described above and possibly introduce a measurement bias. Therefore, it is desirable to damp the pendulum motion of the mass pan. An interesting possibility to damp the mass pan motion is to use sloshing liquids in a sealed ring channel mounted to the mass pan [118, 119].

#### 3.7.4. Alignment of the mass comparator.

For their correct operation, the mass comparator needs to be aligned with respect to vertical. Otherwise, the weighing cell is sensitive to horizontal forces [58]. This sensitivity can be used to align the weighing cell, see [117]. So far two NMIs have built systems with mass comparators, METAS [58] and BIPM [112]. Three more NMIs are planning on using mass comparators, KRISS, NIM, and UME.

#### 3.7.5. Pressure effects.

At a room temperature of 22 °C and an atmospheric pressure of 100 kPa, the density of air is approximately 1.2 kg m^−3^ and the density of a silicon mass standard (one of the lowest density mass standards) is 2300 kg m^−3^. The correction that must be made to the measured mass to allow for its buoyancy is over 500 parts in 10^6^. This correction is difficult to make accurately because the density of the air is dependent on its temperature, pressure and composition. By reducing the air pressure to below 0.1 Pa, the buoyancy correction is much less than 1 part in 10^9^. Most Kibble balances are operated at such pressures to ensure that both the buoyancy and refractive index corrections (section 3.5.2) are negligible.

The reduction in pressure also effects the surface films on the mass and the resulting changes in mass are time dependent and may exhibit hysteresis with variations of pressure. This effect has been extensively investigated [120, 121].

#### 3.7.6. Magnetic forces on the mass.

All mass standards, including platinum–iridium and stainless steel, have a finite magnetic susceptibility [122, 123] which can affect their apparent weight when in the spatially varying magnetic field of a Kibble balance. If the effect cannot be shown to be negligible, it will require correction which can increase the uncertainty of the measured mass. This has been addressed in two ways. Many recent Kibble balances use magnets having a closed magnetic circuit which reduces both the stray field and its gradient thereby reducing the effect considerably. Also research has been carried out to find materials with low magnetic susceptibilities which have the correct mechanical properties to make excellent mass standards. The application of both of these techniques can reduce corrections for the magnetic susceptibility to much less than 20 parts in 10^9^.

#### 3.7.7. Load locks and mass exchangers.

The Kibble balance operates under vacuum and it takes many hours for a freshly pumped balance to stabilise. The pumping can produce temperature changes in the magnet and the moving parts of balance outgas at different, but slowly reducing, rates both of which disturb the weighing measurements. If the balance has to be opened every time the working mass has to be changed, much time can be lost via this mechanism. Some Kibble balances [22] now incorporate mechanisms for storing a number of working masses inside the vacuum chamber and provide mechanisms for loading a selected mass into the balance. Such a mass exchanger allows many comparative investigations to be carried out relatively rapidly.

It is also possible to fit Kibble balances with load locks which allow masses to be introduced into the balance chamber and stored on the mass exchanger. This allows a large number of masses to be measured by the Kibble balance in an efficient manner which is a great advantage for routine operation. The NIST Kibble balance is fitted with both a mass exchanger [124] and a load lock to aid its use in maintaining national and international standards of mass. Both of these are likely to become far more common features of Kibble balances in the near future.

### 3.8. Alignment of the Kibble balance

All existing Kibble balances require precise alignment of both the magnet and the coil [125–127]. The arguments in section 2.1 show that, for balances which have a common weighing and moving mechanism, the alignment requirements may be relaxed, due to the cancellation of the effects of the derivatives of the flux with respect to the non vertical directions [15]. The amount of this relaxation depends on the details of their mechanical construction. If the guidance of the coil is flexible, such that horizontal torques and forces on the coil cause it to move significantly, then the coil needs to be aligned to suppress such movements. This ensures that the theory in section 2.1 applies correctly, and the alignments are carried out using the techniques described below [128]. An alternative is to guide the coil using elements which are stiff enough to suppress motion and the effects of forces and torques in all but the weighing direction. If the position and orientation of the coil can be completely described in terms of the vertical position of the mass pan then, during manufacture, it may be possible to align the balance sufficiently well and operate the balance in a way which eliminates the need for the regular, extremely precise, alignments described below.

#### 3.8.1. Alignment of the magnetic field.

The axis of the magnetic field should be aligned to be vertical but the required accuracy of this alignment varies considerably. For most balances, any misalignment will be compensated by an opposing change in the direction of the axis of the coil to ensure that the force generated by the coil is vertical at the weighing position. But in moving mode the sensitivity of the coil to angular velocities will change as the coil moves vertically. Limits on the requirements are discussed in [13]. A recent publication by the BIPM group discusses different ways to check the field alignment with a dedicated instrument that combines a rotating tilt meter, a Hall sensor, and capacitive sensors [41].

#### 3.8.2. Alignment of the weighing pan.

The weighing pan should be aligned such that the coil does not tilt or move, when the mass is placed on the weighing pan. Note, this step should be performed without current in the coil. The best way to do this is to lock the balance at the weighing position. Using the mass lift, a mass is placed on the mass pan and the motion of the coil is monitored. Ideally the mass pan and the coil swivel about two independent gimbals. However, due to finite stiffness or misalignment between the mass and coil gimbals, there is a coupling and a movement of the mass pan can create movement in the coil which can be corrected by moving the mass pan gimbal with respect to the coil gimbal. The measurement needs to be current-less, because if the coil carries a current, which is reversed when the mass is added changes in electromagnetic forces and torques can mask the effect.

### 3.9. Horizontal forces and torques

Horizontal forces, *F_x_*, *F_y_* and torques, Γ*_x_*, Γ*_y_*, occur, when the coil is not perfectly aligned with the magnetic field. Horizontal forces are caused by an angular misalignment of the field with respect to the coil. The presence of torques implies that the line of action of the force produced by the coil does not pass through the vertical line linking the centre of mass with the point of suspension of the coil. One of many solutions to this is to make the symmetry axis of the coil and the symmetry axis of the magnet coincident with the line mentioned above.

The best way to infer these parasitic forces and torques is by using one or more flexible elements in the coil suspension. These elements convert the forces and torques into linear and angular displacements, which can be measured. The details and the restoring forces and torques depend on the exact design of the coil suspension.

Two coil suspensions are popular. The NIST and NPL Kibble balances use a coil suspension similar to the operating cross of a string puppet. The controlling cross is always parallel to the coil, but both can tilt together with respect to the horizontal plane. In addition, the coil can displace horizontally with respect to the controlling cross in a so called shear motion. The shear motions are measures of the horizontal forces on the coil, the tilt motions are measures of the torque. In the LNE Kibble balance [129] and later the NRC Kibble balance, the coil is suspended from vertically-separated, double gimbals. The angular excursions of the upper gimbals is exclusively given by the horizontal forces on the coil, the excursions of the lower gimbals by a combination of the horizontal forces and the torques on the coil. By measuring four quantities (two in each of the direction *x* and *y*) the forces and torques can be inferred [129]. Typically, the angles of the lower gimbals and the horizontal displacements of the coil are measured.

Various techniques are employed [130] to measure the angular and linear displacements. An autocollimator, an optical lever [131] or a differential interferometer can be used to measure angular displacements. Linear displacements can be measured by reflecting a vertical laser beam from a corner cube and monitoring the position of the reflected beam on a position sensitive detector. Capacitive sensors, reflective optical sensors and interferometers can also be used to detect horizontal motions. Typically, the techniques used can sense linear motion to a fraction of 1 μm and angular motion to about 1 μrad. To convert these sensitivities into force and torque units the stiffness parameters of the coil suspension are required. These should be given in the publications describing each Kibble balance.

**Horizontal forces** arise if the coil is tilted with respect to the magnetic field. In the alignment procedure the current in the coil is altered and the horizontal displacement of the coil is monitored. If the horizontal displacement, and by implication the associated horizontal force, is too large, the tilt angle of the coil is changed and the process is repeated until the horizontal forces have been suppressed to the level desired. If the suspension of the coil is such that it can tilt freely, then the easiest way to change the tilt is to add a mass on the coil or a connected mechanical structure and the coil will tilt into a new equilibrium position.

**Torques** on the coil are created when the coil is not centred in the magnetic field, more specifically, when the centre of mass of the coil and the magnetic centre of the coil magnet system are horizontally displaced. To measure the torques, the current in the coil is reversed and the corresponding coil motion recorded. The torque on the coil can be minimized by three means, (a) the coil can be displaced, (b) the magnet can be displaced, or (c) masses on the coil can be moved to change the mass centre of the coil. Typically, the third option typically changes the tilt of the coil and should be avoided.

#### 3.9.1. Parasitic motions in moving mode.

In moving mode, the coil should follow the ideal trajectory given by the mechanical system, e.g. a perfect vertical motion for the wheel balance or an arc-shaped course for a beam balance. Velocities present during the moving phase that are not explained by the ideal system are parasitic motions and stem from minor deviations of the real mechanical system from the ideal system. Very often these deviations can be trimmed away. In most cases, the ideal motion of the coil does not include any horizontal or angular velocities of the coil. Ideally the coil should translate without changing its roll, pitch, or yaw. Any angular velocity is a parasitic motion.

The parasitic velocities are measured by the same detectors and instruments that sense the parasitic forces, see section 3.9.

**Horizontal velocities** occur due to small misalignments in the guiding mechanism. The root cause depends on the detailed guiding mechanism used in each Kibble balance and it is therefore impossible to give a general description of the methods used to minimize these velocities. For example, in the classic beam balance, one horizontal velocity is given by the angle of the flat with respect to the horizontal plane. By tilting the flat, the horizontal velocity can be changed and eventually minimized.

An easy way to measure horizontal displacement is the use of a vertical laser beam directed into a corner cube mirror mounted on the coil. The reflected beam is monitored with a position sensitive detector. It will move twice the distance that the corner cube has moved. With this technique it is possible to resolve horizontal motions below 1μm. Note, the laser beam has to be vertical, otherwise the motion of the coil is optimized for the direction of the laser beam and not local vertical. The same techniques described to align the main interferometer to vertical, see section 3.5.5, can be used for this beam. Ideally, the interferometer beam can also be utilized for this measurement, minimizing the number of beams that have to vertically aligned.

**Angular velocities** of the coil in the moving mode have to be minimized, as well. One culprit for such parasitic rotations are the wires that connect the coil to the electronics. Because of the motion of the balance, the torque produced by these wires changes. If the coil suspension is compliant to torque changes, the coil will rotate. Care must be taken in routing the wires such that the torque produced by them is small and, ideally, constant over the velocity sweep. This can be done by using thin wires at small lever arms. The wires can be made softer by heat treating them. In the NIST Kibble balance, the coil can rotate around the vertical axis. This degree of freedom is not compliant in most other watt balances. To eliminate parasitic rotation around the vertical axis in the weighing and moving mode, the NIST researchers employ a feedback system that produces an electrostatic torque on the coil suspension that keeps the coil at constant azimuthal angle.

### 3.10. Verification

A Kibble balance is a complex, automated measuring instrument and it can be difficult to diagnose problems and verify its correct operation if it is treated as a monolithic device. Problems of diagnosis and verification can be simplified by the technique of splitting the watt balance into a number of subsystems which can be tested individually. However, it is important to ensure that the instrument can be split up without changing the characteristics of its parts. For example, if the correct operation of one part is being disturbed by the operation of another via an unexpected route, both parts may work perfectly when separated but their operation may be affected in subtle ways when both parts are installed to the apparatus. Some ways of minimising such effects are discussed in section 3.11.4. In the following sections, it is assumed that the parts have been designed to minimise unwanted interactions and tests for such effects have been carried out where feasible.

#### 3.10.1. Subsystem verification.

The Kibble balance can be broken into a number of independent subsystems. In many cases, the correct operation of these subsystems can be verified independently of the main apparatus.

##### Resistor

The resistor must be measured against a QHR and this can be used to verify the stability of the resistor as described in section 3.4.2.

##### Josephson voltage reference

This is often verified by comparison against another Josephson voltage reference [53, 132–134].

##### Voltmeter

The operation of the voltmeter can be checked using the Josephson voltage reference.

##### Laser

This can be verified by comparison with an iodine stabilised laser.

##### Time reference

Verification of the time reference was described in section 3.5.8.

##### Software

The software for the system should be subject to tests. Individual software subsystems can be checked independently but it is advantageous to be able to test the whole system using synthetic measurement data either with or without synthetic noise. This requires considerable effort especially as gravitational corrections are time dependent and must be synthesised correctly. However, if the data is synthesised at the lowest level of the system i.e. at the level of voltmeter and interferometer output data, it can be used to validate the whole of the data processing system. This can include checks on the corrections which are applied to the results either at the time of data acquisition or during post processing.

#### 3.10.2. System verification.

Many parts of the system can be verified by setting carefully chosen parameters to values outside their usual range and by checking that the effect of the change is as expected [14, 70].

The linearity of the system can be checked by weighing masses of differing values and by making measurements at differing coil velocities. This is a necessary, but not sufficient, test for the accuracy of the apparatus because offsets which are proportional to the quantity being changed will not be detected [48].

The ultimate test of a Kibble balance is the comparison of its results with those of independent balances. This should be carried out in the manner of a formal comparison [135] so that any problems that are discovered are properly identified and corrected. A comparison of different Kibble balances and of the two Avogadro spheres was carried out in 2016 [136] to check for consistency before the revision of the international system of units. Such comparisons will form the basis of the global mass scale which needs to be derived from a large number of independent primary measurements.

### 3.11. Environmental effects

A Kibble balance will always be sensitive to some environmental effects for example: the conversion between force and mass depends on the free-fall acceleration *g* which is dependent on the position of the mass within the apparatus and atmospheric pressure outside the laboratory to name but two. However, the aim of the design of a Kibble balance should be to minimise the the effect of the environment on the balance.

#### 3.11.1. Ground vibration.

Ground vibration can affect both weighing and moving phases, as described in section 3.3. If critical parts of the balance are constructed carefully to ensure that the vertical velocity measurement is highly correlated with the voltage produced by the coil, the effects of ground vibration in the moving phase can be greatly reduced but is difficult to eliminate them entirely. In common with precise mass balances, most practical Kibble balances would benefit from being sited in an area with low ground vibration.

Anti-vibration systems can be used to reduce the effects of ground vibration but care has to be taken to ensure that the angular stability of the anti-vibration system is sufficient to minimise the overall noise of the balance.

#### 3.11.2. External magnetic fields.

Kibble balances are usually sited far from significant sources of magnetic fields; therefore, the remaining sources of magnetic interference are: changes in the magnetic field of the earth and local line frequency interference. The sensitivity of the Kibble balance to these effects is strongly dependent on the design of the magnet. As described in section 3.2, most recent balances use variants of the closed magnetic circuit design introduced by the BIPM. This design provides good rejection of both changes in the magnetic field of the earth and local line frequency fields. However, it is good practice to ensure that line frequency fields are minimised by ensuring that no mains wiring loops encircle the room containing the balance. For magnets which are more sensitive to external fields, a sensitive magnetometer and Helmholtz coil can be used to null temporal changes in the field of the earth as described in [48].

#### 3.11.3. Temperature effects.

As described in section 3.2, some Kibble balances use magnets with temperature coefficients of approximately −400 parts in 10^6^ · K^−1^ which, even with vacuum isolation, required local temperature control at the level of ±4 mK [48]. As described in section 3.2, recent designs have reduced this considerably but Kibble balances still need sufficient temperature control to eliminate noise and uncertainty caused by temperature gradients which can cause changes in the arm lengths of the balance and variations of thermal EMFs in critical parts of the measurement circuit.

#### 3.11.4. Shielding and electrical isolation.

The Kibble balance is a complex electrical measuring instrument which must measure its principal electrical quantities with uncertainties approaching 1 part in 10^9^. To simplify the task of ensuring and verifying that the measurement achieves these uncertainties, it is advantageous to isolate the principal electronic instruments of the measurement system to ensure that currents can only flow through the system in a predictable way. This is achieved by placing the entire apparatus in an electrostatic shield and ensuring that each instrument is similarly shielded and that there is a high level of both dc and ac isolation between each instrument, the mains, and the controlling computer. This eliminates uncontrolled ac or dc currents flowing between instruments, through critical parts of the measurement system, via unintentional leakage paths to either the controlling computer or the mains. Very high levels of isolation (greater than 10 TΩ and leakage at line frequency less than 1 pA) can be achieved, for example, by using the techniques described in [67, 137].

## 4. Existing implementations and their results

At the time of this writing (spring of 2016) researchers at five laboratories have published results with Kibble balances and researchers at one laboratory have published two results with a joule balance, that has been substantially altered for the second publication. Several laboratories are currently in the process of designing or building a Kibble balance. At four laboratories (METAS, NPL, NIM, and NIST), more than one balance have been built. To distinguish the results from different iterations, we assign an incremental version number to the Kibble balance at a given institute. However, one has to be careful with this nomenclature. Typically, Kibble balances are constantly improved and two results are rarely published with exactly the same instrument. Very often the hardware is changed, sometimes the alignment procedures, the measurement protocol, or the data analysis. Assigning a new version number to each of these incremental changes would lead to an inflation of version numbers and would ultimately render them meaningless. We assign a new version number only when substantial changes were made. Examples for substantial changes are a redesigned magnet system or the addition of a vacuum chamber. The early experiments were performed in air and vacuum chambers were added later on.

Table 4 lists the joule and Kibble balances that have been described in the metrological literature in the past 27 years. The table has 16 rows describing balances in all stages of completion, from the planning stage to completely dismantled. Eleven numerical values have been produced.

Some of the results listed in table 4 can be discarded because new results from the same instrument have superseded older results. One example is the NPL-2 Kibble balance. This balance was transferred to the Canadian Metrology Institute (NRC) in 2009. A combination of two systematic effects was discovered before the system was due to be shut-down and dismantled. Unfortunately, there was not enough time to carefully estimate the bias that these systematic effects introduced into the result. As a consequence, the uncertainty budget had to be significantly increased from the originally estimated uncertainty of 36 parts in 10^9^ to 200 parts in 10^9^. Upon arrival in Canada, these effects were carefully studied and the corresponding entries in the uncertainty budget were considerably reduced. Hence, the NRC result can be considered to effectively supersede the NPL result.

In 2014, due to the extraordinary circumstances of the possible redefinition of the kilogram in 2018, the International Prototype of the Kilogram was taken out of the vault and measured against the witnesses, the working standards at BIPM, and several national prototypes. During this extraordinary verification, an offset between the working standards at the BIPM and the IPK was found [143–145]. It is now believed that the use of a particular balance, with automatic mass exchange, caused small amounts of wear on the working standards thereby reducing their mass. Hence, a difference of 35 μg between the mass unit as maintained by the BIPM and the mass unit represented by the IPK was found in 2014. After a careful analysis of the data, it could be shown that this bias started building up from 2003 to about 2014. As a consequence, the calibration of all masses in this time interval had to be corrected. NIST and NRC have published corrections to their measurements of *h* using newly calculated SI values of their working masses [139, 141]. Table 4 shows the latest number, including the mass correction.

**Figure 5.**
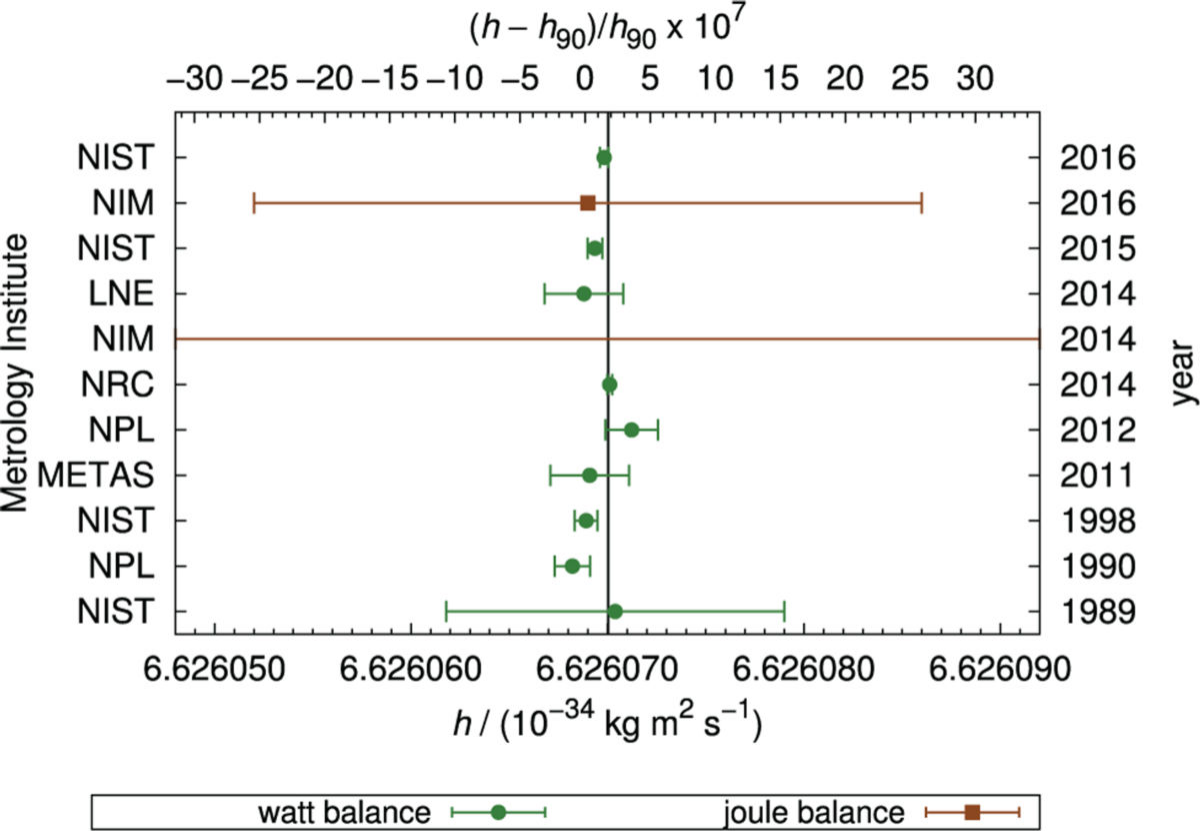
Results of published results obtained by Kibble and joule balances. The abbreviations on the left give the institution where the experiments were performed. The numbers on the right indicate the years the results were published. The dark vertical line is the value of *h* as recommended by CODATA in its 2014 adjustment [142]. The error bars denote the one sigma uncertainties given by the researchers in their respective publications. See table 4 for references.

**Figure 6.**
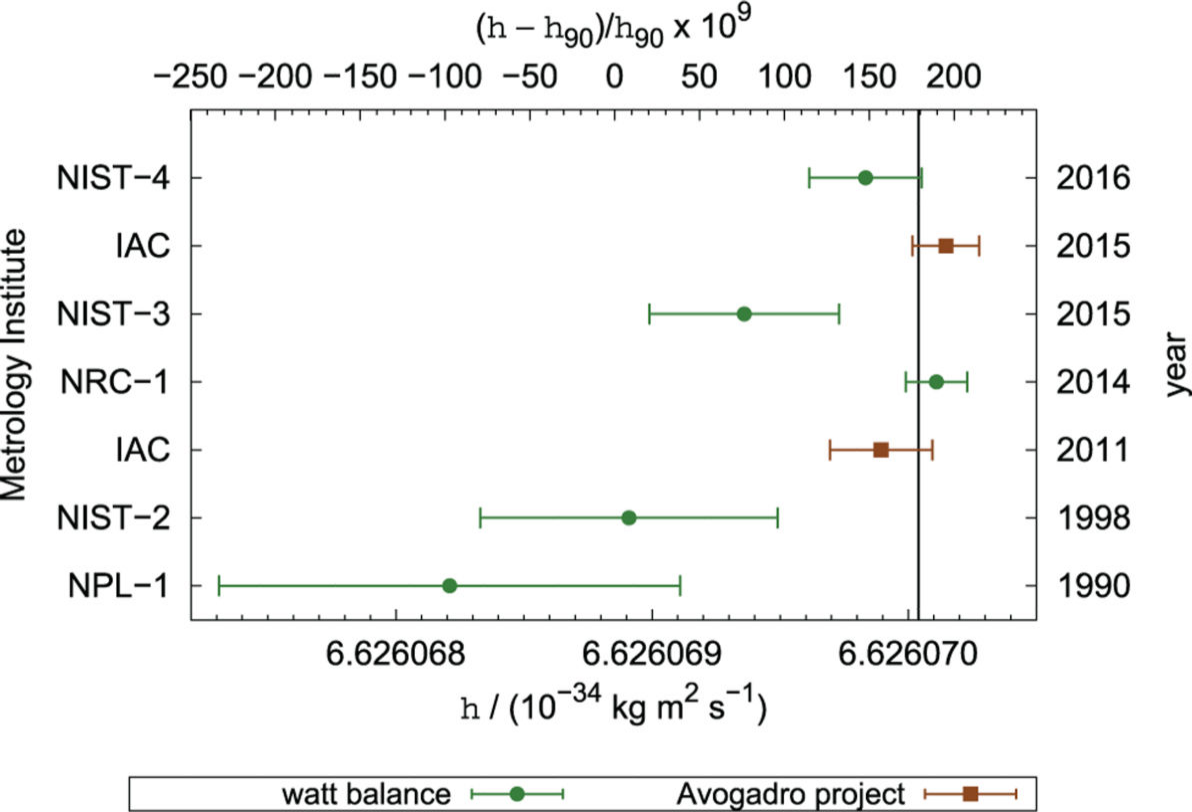
Measurements of *h* with relative standard uncertainties below 2 × 10^−7^. The dark vertical line is the value of *h* as recommended by CODATA in its 2014 adjustment [142]. The uncertainty bars show the standard uncertainty of each result. See table 4 for references.

Figure 5 shows the eleven results listed in table 4. The horizontal scale is large making it difficult to compare the most precise measurements with each other and the recommended value by the Task Group on Fundamental Physical Constants under the auspices of the Committee on Data for Science and Technology (CODATA) [142]. Figure 6 shows the measured values of *h* with relative standard uncertainties below 2 × 10^−7^. This threshold was chosen, because it was used by the working group on the realization of the kilogram (WGRkg) within the Consultative Committee for Mass and Related Quantities (CCM) as the largest acceptable relative uncertainty to participate in the pilot study. Included in the plot are also the two most recent results published by the International Avogadro Collaboration (IAC). There are a total of seven *h* values with relative standard uncertainties below 2 × 10^−7^.

While the scatter in figure 6 is still large, the figure clearly demonstrates the improvements in uncertainties in the best experiments over the past 27 years. It also seems that the results from earlier Kibble balances tend to be lower than the present results although there is no obvious explanation for this.

The seven most precise values agree reasonably well. More importantly, the results measured with Kibble balances agree with results published by the International Avogadro Collaboration. Researchers at five other laboratories are constructing Kibble balances and their results are expected in the next few years. The researchers at NIM are working on a modified version of the joule balance which will use a mass comparator for weighing. When these instruments are operational, it will increase the number of instruments capable of maintaining the mass scale in the future.

## 5. Future directions

Even after about 40 years [146–149], the Kibble balance field is still progressing. Improvements are being made regularly and there is no evidence to say that such improvements will stop after the 2018 redefinition of the kilogram. In fact, the ability to relate the definition directly to measurements of mass and force at many different scales may drive further innovations.

### 5.1. Improvements to existing techniques

Some improvements will be made by the painstaking identification of problem areas or by careful theoretical analysis of possible sources of uncertainty [150–155]. However, some improvements may come about by technological improvements in related areas which will allow measurements critical to the Kibble balance to be carried out either more easily or with lower uncertainties. A few possible improvements are listed below.

- Using simplified Kibble balance designs at all scales.
- Making position and velocity measurements using optical comb based interferometers.
- The development and use of better absolute gravimeters, both conventional and atomic, to reduce the uncertainty due to the gravimeter.
- Using Josephson junction array voltage references made with High TC materials, which work at liquid nitrogen temperatures, to reduce the cost of operating the apparatus.
- Using Josephson voltmeters [156] to simplify the voltage measurements.
- The use of graphene based QHR systems for local measurement of the working resistors.
- Direct operation against QHR devices and QHR arrays which would eliminate the need for a resistance measurement system.

### 5.2. Extension to smaller and larger masses

The Kibble balances discussed above operate at the mass range from 100 g to 1 kg. This section discusses the extension of the technique to mass values outside this range.

**Figure 7.**
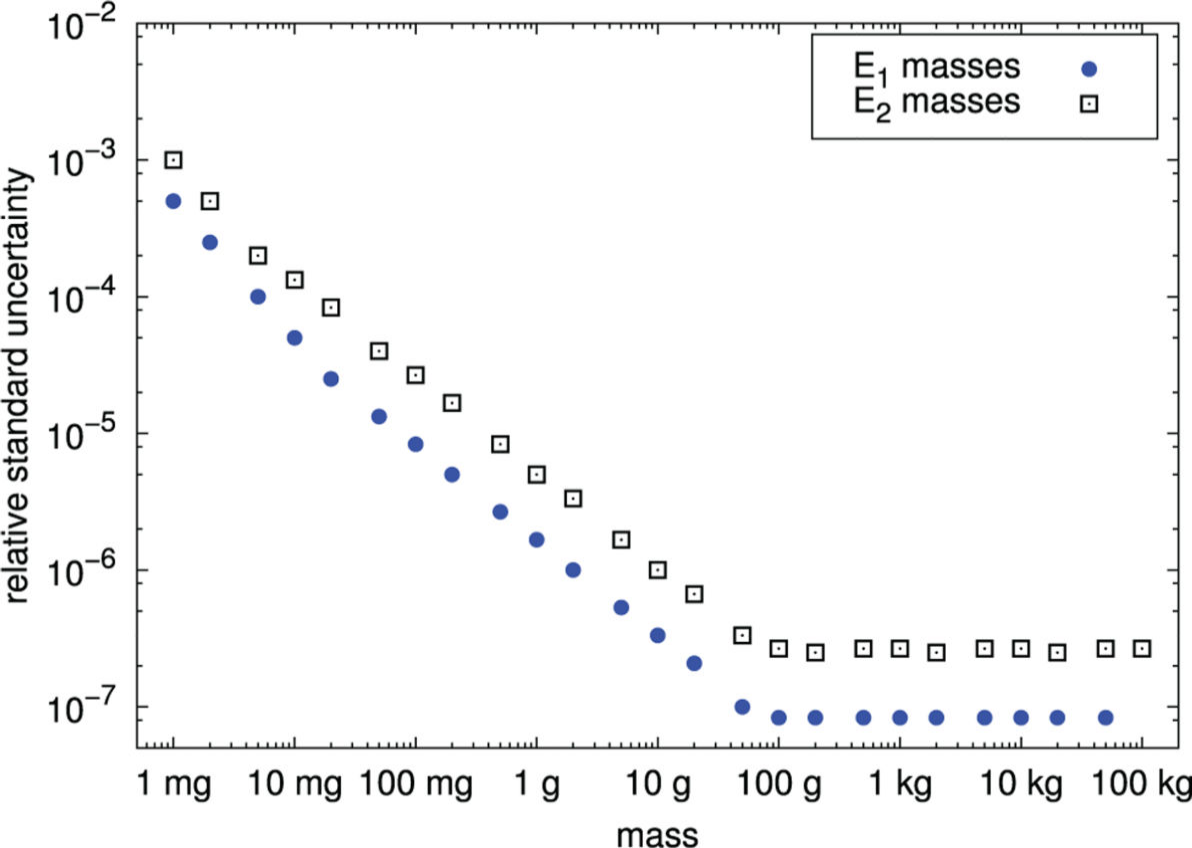
The required relative standard uncertainties for *E*_1_ and *E*_2_ weights as a function of the nominal mass value according to [157].

Two design parameters need to be considered when planning a Kibble balance for the purpose of realizing the unit of mass, (1) the mass value and (2) the desired relative standard uncertainty. The existing system of mass dissemination can be used to provide guidance on the standard uncertainty as a function of mass value. Figure 7 shows the required relative uncertainties for weighing *E*_1_ and *E*_2_ masses according to the recommendation of the Organisation Internationale de Métrologie Légale (OIML) [157]. According to this OIML recommendation, calibration weights are divided into two different classes: *E*_1_ and *E*_2_. The primary use of weights in the class *E*_1_ is to calibrate *E*_2_ weights. These are used to calibrate weights of lesser quality and weighing instruments of special accuracy class I.

Figure 7 shows that the relative standard uncertainty is independent of the mass value for nominal values larger than 100 g. This makes it difficult to justify a Kibble balance above 1 kg, because it seems possible to disseminate multiples of a mass value of 100 g with little loss of relative uncertainty. So, there is little reason to realize the unit of mass at higher values, unless a much better uncertainty than at the 1 kg level can be achieved or that there are other reasons for realising mass or force at such levels without the need for a calibration mass. Reducing the uncertainty seems difficult, because one has to produce a much larger electromagnetic force to balance larger weights. A larger electromagnetic force requires either more current in the weighing mode or more turns on the coil. Both measures will produce more magnetic flux in the coil during weighing mode and also more ohmic heating. Both effects will result in non-linear effects in the active magnetic material and the yoke. The biases and uncertainties associated with this larger disturbance will quickly dominate the uncertainty budget.

The uncertainty situation is different for mass values below 100 g. Here, in the current mass dissemination, the relative uncertainty increases with decreasing nominal value. A Kibble balance with smaller nominal value and sufficient complexity should be able to maintain an uncertainty of a few parts in 10^8^ down to 1 g. This complexity comes with a price that may not be warranted for a 1 g mass realization. But, a Kibble balance that can measure masses between 1 g and 100 g with a relative standard uncertainty of 1 part in 10^6^ provides interesting benefits and can even revolutionize weighing technology on the factory floor. This uncertainty level is competitive with the uncertainties achieved with *E*_1_/*E*_2_ weights. However, the Kibble balance could be used to weigh masses without having to use calibration weights. This could shorten the traceability chain and be more cost effective. For the Kibble balance technology, many uncertainties become much easier at this uncertainty level. For example, most of the gravitational corrections discussed in section 3.6 can be ignored.

For mass values in the region of 10 mg and below, the electromagnetic Kibble balance principle becomes more difficult to apply and the electrostatic Kibble balance [158] is better suited. The size of the geometric factor in an electromagnetic Kibble balance is driven by two competing requirements. For the velocity mode, the geometric factor should be large to maximize the induced voltage for a given velocity. In the weighing mode, the current is inversely proportional to the geometric factor and a smaller geometric factor is beneficial because it requires a large current that can easily be measured. The ideal geometric factor is a compromise of these two considerations. Since the velocity mode is independent of the test mass, decreasing the geometric factor will lead to an increase in relative uncertainty assuming the system has been optimized for minimal uncertainty. However, leaving the geometric factor the same will also increase the relative uncertainty. In this case, the required current will get disproportionately smaller with smaller masses. Precisely measuring these currents will be limited by noise in the current source, thermal and Johnson noise in the resistor, and resolution. Regardless if the geometric factor is decreased or not, building electromagnetic Kibble balances for smaller nominal masses will lead to an increase in relative uncertainty and at some point the uncertainties obtained with such Kibble balances may no longer be competitive with those from electrostatic balances. Electrostatic balances, which use capacitance measurements, have been in use for many years [159] but similar balances using the principles of the electrostatic watt balance [16] have not yet been built, but offer an alternative.

## 6. Conclusion

The Kibble balance is an instrument for relating mass and force to fundamental and atomic constants. Within the limitations described in section 5.2, it is capable of measuring over a continuous range of masses or forces with little change in its measurement uncertainty. This ability offers considerable advantages to laboratories who wish to realise a range of masses directly from the definition of mass within the new SI.

At present, the efforts of Kibble balance groups are concentrated on measuring the value of the Planck constant, in terms of the existing definition of the kilogram with the lowest possible uncertainty. Once the redefinition of the SI has taken place the emphasis will change to maintaining national and international mass scales [160]. In this endeavour, we have no doubt that further advances will be made to the Kibble balance technique to make it simpler, more accurate and wider ranging.

The true power of fixing *h* in the new SI is that it enables a range of techniques to realise SI mass from Kibble balances and x-ray crystal density techniques [161] at the macroscopic level to Compton clocks [162] at the atomic scale. No one technique ‘defines’ mass but they can all measure SI mass, at an appropriate scale, to the best of their abilities. In this way, improvements to any valid technique, which can relate mass and *h*, are never held back by the need to relate results to an artefact standard at another scale.

## Figures and Tables

**Table 1.** Comparison of the magnet design of Kibble balances at nine different laboratories.

Laboratory	*B*_g_ (T)	*g*_w_ (mm)	*g*_h_ (mm)	*r*_c_ (mm)	$\frac{\partial \text{Φ}}{\partial z}$ (T m)	Type	Reference
BIPM	0.6	13	80	125	≈ 500	BIPM	[41]
KRISS	0.73	25	60	208	462	BIPM	[17]
LNE	0.95	9	60	134	536	LNE	[40]
METAS	0.64	8	50	100	757	BIPM	[42]
MSL	0.6	16	100	120	420	MSL	[43]
NRC	0.42	24	102	170	300	NPL	
NIST	0.55	30	80	215	710	BIPM	[44, 45]
UME	0.55	10	34	72	1250	BIPM	[34]

*Note*. The magnet designs of the latest Kibble balances as of spring 2016 are shown. The column labelled *B*_g_ gives the magnetic flux density along the radial direction in the centre of the gap with a gap width *g*_w_ and a gap height *g*_h_. The mean coil radius is abbreviated with *r*_c_.

**Table 2.** Types of interferometers used in the latest Kibble balances built by different laboratories.

Laboratory	Mode	Laser	Type
BIPM	Heterodyne	Nd:YAG	Michelson
KRISS	Homodyne	I2	Michelson
LNE	Heterodyne	Nd:YAG	Michelson
METAS	Homodyne	HeNe	Fabry–Perot
MSL	Heterodyne	HeNe	Michelson
NPL	Homodyne	I2	Michelson
NRC	Homodyne	HeNe	Michelson
NIST	Heterodyne	Nd:YAG	Michelson

**Table 3.** Balances at nine different laboratories.

Lab.	Design	Reference
BIPM	Mass comparator	[112]
KRISS	Mass comparator	[17]
LNE	Flexure balance	[113]
METAS	Mass comparator	[22]
MSL	Pressure balance	[43]
NIM	Beam bal./mass comp.	[114]
NRC	Beam balance	[70]
NIST	Wheel balance	[81]
UME	Mass comparator	[34]

**Table 4.** List of published and ongoing joule and Kibble balance research.

Lab.	Ver.	$h/\left(10^{-34}\text{J s}\right)$	$\frac{\sigma_{\text{h}}}{h}/10^{-9}$	$\left(\frac{h}{h_{90}}-1\right)/10^{-9}$	Year	Reference	Comments
BIPM	1					[32]	Undergoing improvements.
KRISS	1					[17]	Under construction.
LNE	1	6.626 0688	302	−8	2015	[138]	First result in air, ongoing.
METAS	1	6.626 0691	302	37	2011	[21]	Final result.
METAS	2					[23]	Ongoing.
MSL	1					[43]	In design.
NPL	1	6.626 068 21	136	−97	1990	[5]	In air, non radial field.
NPL	2	6.626 071 23	200	359	2012	[48]	Systematic found, before sending to NRC.
NRC	1	6.626 070 11	19	189	2014	[139]	IPK correction applied, ongoing.
NIM	1	6.626 104	8900	5300	2014	[28]	Air coil system.
NIM	2	6.626 069	2566	22	2016	[114]	Iron free permanent magnet system.
NIST	1	6.626 070 39	1300	232	1989	[9]	Solenoid to generate field, in air.
NIST	2	6.626 068 39	87	8	1998	[140]	Superconducting solenoid, in air.
NIST	3	6.626 069 36	57	77	2015	[141]	In vacuum, IPK correction applied.
NIST	4	6.626 069 83	34	148	2016	[81]	Permanent magnet, ongoing.
UME	1					[34]	In planing.

*Note*. Several institutes have worked on different versions of the Kibble balance. If a result has been published the number can be found in the third column. This is the latest number obtained from the version of the balance indicated in the second column. The result is published in the reference indicated in the fifth column.
